# Transformation of Iodosulfuron-Methyl into Ionic Liquids Enables Elimination of Additional Surfactants in Commercial Formulations of Sulfonylureas

**DOI:** 10.3390/molecules26154396

**Published:** 2021-07-21

**Authors:** Witold Stachowiak, Radosław Szumski, Jan Homa, Marta Woźniak-Karczewska, Anna Parus, Beata Strzemiecka, Łukasz Chrzanowski, Michał Niemczak

**Affiliations:** Department of Chemical Technology, Poznan University of Technology, 60-965 Poznan, Poland; witold.stachowiak@doctorate.put.poznan.pl (W.S.); r.szumski@isofcik.eu (R.S.); jan.j.homa@doctorate.put.poznan.pl (J.H.); marta.wozniak-karczewska@put.poznan.pl (M.W.-K.); anna.parus@put.poznan.pl (A.P.); beata.strzemiecka@put.poznan.pl (B.S.); michal.niemczak@put.poznan.pl (M.N.)

**Keywords:** HILs, weed killer, plant protection, sulfonylurea herbicide, ALS inhibitors, volatility, soil mobility, stability

## Abstract

Efficient use of herbicides for plant protection requires the application of auxiliary substances such as surfactants, stabilizers, wetting or anti-foaming agents, and absorption enhancers, which can be more problematic for environment than the herbicides themselves. We hypothesized that the combination of sulfonylurea (iodosulfuron-methyl) anion with inexpensive, commercially available quaternary tetraalkylammonium cations could lead to biologically active ionic liquids (ILs) that could become a convenient and environment-friendly alternative to adjuvants. A simple one-step synthesis allowed for synthesizing iodosulfuron-methyl based ILs with high yields ranging from 88 to 96% as confirmed by UV, FTIR, and NMR. The obtained ILs were found to possess several favorable properties compared to the currently used sodium salt iodosulfuron-methyl, such as adjustable hydrophobicity (octanol-water partition coefficient) and enhanced stability in aqueous solutions, which was supported by molecular calculations showing cation–anion interaction energies. In addition, soil mobility and volatility of ILs were more beneficial compared to the parental herbicide. Herbicidal activity tests toward oil-seed rape and cornflower revealed that ILs comprising at least one alkyl chain in the decyl to octadecyl range had similar or better efficacy compared to the commercial preparation without addition of any adjuvant. Furthermore, results of antimicrobial activity indicated that they were practically harmless or slightly toxic toward model soil microorganisms such as *Pseudomonas putida* and *Bacillus cereus*.

## 1. Introduction

Sulfonylureas are a class of compounds used primarily as herbicides in agriculture or as antidiabetic drugs in medicine [1]. The unique herbicidal activity of sulfonylureas was discovered in 1975 and soon this new class of agrochemicals was rapidly commercialized, largely due to the extremely low doses of active ingredient needed for weed control (corresponding to approx. 10–40 g of chosen compound per hectare) [2]. It should be noted that the recommended doses of glyphosate or phenoxyacetic acids such as 2,4-dichlorophenoxyacetic acid (2,4-D) [3] or 4-chloro-2-methylphenoxyacetic acid (MCPA) [4] are higher (approx. 500–1500 g per hectare).

**Figure 1:**
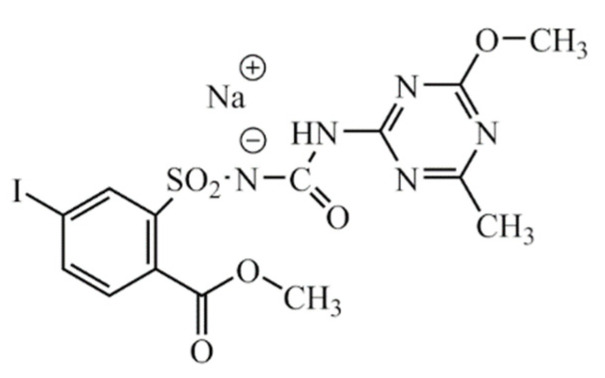
Structure of iodosulfuron-methyl sodium salt.

Sulfonylureas are known as selective herbicides, and are extremely effective against abroad spectrum of weeds, e.g., ryegrass, oats, wild radish, fireweed, toad rush, legumes, and a number of other troublesome weeds [5,6,7]. Sulfonylureas are absorbed by the above-ground parts of plants (LIT), and their mechanism of action involves inhibition of the enzyme acetolactate synthase (ALS), which is involved in the biosynthesis of the branched-chain amino acids (BCAAs)—e.g., leucine, valine, and isoleucine [8]. All the above-mentioned features of this class of herbicides make them very popular among farmers. More than 40 sulfonylurea-based compounds have been successfully commercialized since the 1990s. In France, for example, 53 tons of sulfonylurea-based herbicides were used in 1995, and that number has increased nearly fivefold to 246 tons in 2018, while, in Germany, 381 tons were used in 2015. Overall, trends indicate a steady increase in demand for this type of herbicides worldwide [9]. Currently, one of the most effective sulfonylurea-based herbicides globally is sodium salt of iodosulfuron-methyl presented in the Figure 1 [10].

Years of intensive use of sulfonylurea herbicides have revealed some of the problems associated with their commercially used formulations. Most sulfonylureas exhibit some stability problems and may degrade during long-term storage and transportation, or precipitate out of solution [11,12,13]. Therefore, various substances, so-called adjuvants, need to be introduced into commercially available formulations to stabilize them [14,15,16,17,18]. Furthermore, the herbicidal efficacy of sulfonylurea herbicides can be substantially improved by using appropriate adjuvant additives [19,20]. Surprisingly, these are not considered as active substances, and therefore the regulatory restrictions on their use are not as stringent as for the herbicides themselves [21]. For this reason, despite their diverse chemical structures and wide applications, the effects of adjuvants on living organisms and the environment have not been routinely and thoroughly investigated [21]. The best-known example demonstrating harmful influence of adjuvants refers to the reported toxicity of the adjuvant polyethoxylated tallow amine (POEA) known as the ingredient of glyphosate-based formulations, like Roundup. Experimental studies suggest that the toxicity of the POEA is greater than the toxicity of glyphosate alone and commercial formulations alone [22]. Commercial sulfonylurea formulations typically contain various adjuvants, such as nonionic surfactants (NIS) or ionic surfactants such as salts of sulfosuccinates and the most commonly—Heavy oil fractions such as naphtalene or naphta [23,24,25].

One method proposed to reduce the use of adjuvants in herbicidal treatments is to convert herbicides to ionic liquids. This concept is based on pairing appropriate organic cation—with herbicidal anion. Herbicidal ionic liquids (HILs) have numerous advantages such as adjustable physicochemical properties e.g., phase transitions (i.e., melting point), viscosity, polarity, water solubility or mobility in soils and waters [7,20,26]. The presence of an ionic bond in their structure reduces the volatility which positively affects the safety associated with their application [27]. Additionally, HILs possessing at least one long alkyl chain can exhibit excellent surface active properties, eliminating the need for additional surfactants, and often allowing herbicide application at lower than normal concentrations [28,29]. The resulting ionic pairs have been shown to exhibit a number of desirable physicochemical and biological properties [28,30,31,32].

Earlier publications reported the synthesis and characterization of HILs containing the following sulfonylureas anions: iodosulfuron-methyl [7], metsulfuron-methyl [33] and nicosulfuron [14]. However, from an economic point of view, new herbicidal formulations should be cost-effective and easy to prepare. Thus, it is preferred to use already commercially available and inexpensive cations instead of preparing new ones in multistep synthesis [7,14,33]. Alkyltrimethylammonium halides and dialkyldimethylammonium halides are popular cationic surfactants that are commonly used in cosmetic, detergents, as fabric softeners, preservatives or in production of organic clays [34,35,36]. Moreover, a positive opinion of the European Food Safety Authority (EFSA) [37] has allowed their use in cosmetic products. Generally, quaternary ammonium compounds are biodegradable under aerobic conditions. However, the sorption process is much faster than biodegradation, so these compounds are susceptible to accumulation in the environment, especially anoxic and anaerobic environments [31,38]. Due to widespread production and good availability, their cost is relatively low compared to other tetraalkylammonium cations.

The main objective of the present study was to verify the hypothesis that introduction of commercially available tetraalkylammonium cations containing at least one long alkyl chain into structure of sulfonylurea-based herbicide can reduce the use of other additives, minimize negative environmental impacts, while maintaining high herbicidal efficacy. The work also includes a thorough evaluation of the effect of alkyl chain length in the cation on the physicochemical properties (i.e., solubility, octanol-water partition coefficient, leaching from soil and stability), biological activity and biodegradability.

## 2. Results

### 2.1. Synthesis

Our hypothesis to potentially eliminate the use of adjuvants, and minimize negative environmental impacts, while maintaining high herbicidal efficacy required the synthesis of new forms of sulfonylurea-based herbicides. Therefore, in the present work, new ionic liquid forms of sulfonylurea herbicides (iodosulfuron-methyl) were synthesized by anion exchange reactions, starting from eight commercially available, inexpensive and widely used quaternary tetraalkylammonium halides (five alkyltrimethylammonium and three symmetrical dialkyldimethylammonium). Among the cations used, two contain an alkyl group of natural origin: cocotrimethylammonium chloride (S1, CAS 61789-18-2) possesses a ‘*coco*’ group that was derived from coconut oil, di(hydrogenated tallow)dimethylammonium chloride (S8, CAS 61789-80-8) contain ‘*hydrogenated tallow*’ substituent, which was rendered from beef or sheep fat. The implementation of such naturally derived cations in the structure of IL may lead to a reduction in their toxicity towards vertebrates compared to fully synthetic cations. As a result, ILs containing such ions may exhibit relatively low acute toxicity (V category) according to the criteria of the Globally Harmonized System of Classification and Labelling of Chemicals (GHS) [4,39]. The other quaternary halides used in the syntheses contained one long alkyl: tetradecyl (C_14_H_29_) group (myristyltrimethylammonium bromide, S2, CAS 1119-97-7), hexadecyl (C_16_H_33_) group (cetyltrimethylammonium chloride, S3, CAS 112-02-7), octadecyl (C_18_H_37_) group (stearyltrimethylammonium chloride, S4, CAS 112-03-8), docosyl (C_22_H_45_) group (behenyltrimethylammonium chloride, S5, CAS 17301-53-0) or two long alkyls: octyl (C_8_H_17_) groups (dimethyldioctylammonium chloride, S6, CAS 5538-94-3), decyl (C_10_H_21_) groups (didecyldimethymammonium chloride, S7, CAS 7173-51-5).

**Scheme 1:**
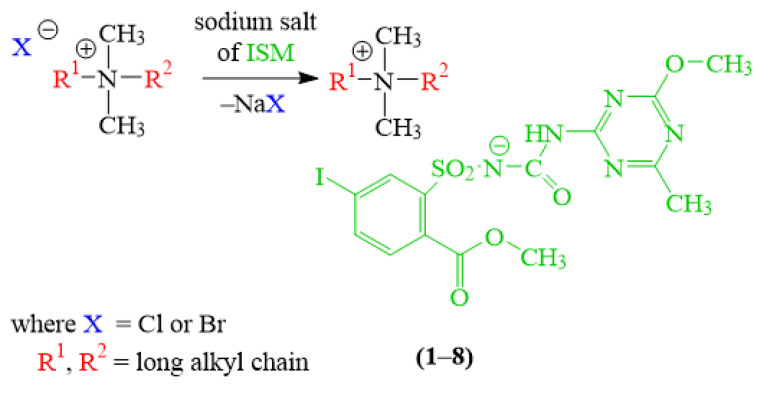
Method of preparation of iodosulfuron-methyl-based salts with commercial tetraalkylammonium cations (**1**–**8**).

The synthesis was carried out in two approaches—The first of which involved the standard laboratory synthesis of HILs, while the second was simplified, which significantly reduced the time and total costs of the procedure. According to the first method, the products were obtained by an ion-exchange reaction between corresponding tetraalkylammonium halide and the sodium salt of iodosulfuron-methyl carried out in water at ambient temperature (40 °C), as shown in Scheme 1. All products were isolated from the reaction mixture by a biphasic extraction technique using a non-polar solvent (chloroform). All compounds were then subjected to thorough drying to eliminate the presence of any of the solvents used. Our second proposed synthesis method involves ion exchange in short chain alcohols, such as methanol or ethanol. It is not only as efficient compared to the traditional one, but also much faster and more environmentally friendly. However, it should be noted that these products usually contain an inorganic salt. In the case of ethanol the amount of NaCl in the product was about 0.5%, while in methanol it reached up to 2.5%. Nevertheless, such amounts of NaCl in agrochemicals, do not pose any threat to environment as well as to cultivated crops, so this method is substantially advantageous in terms of production on commercial scale. In additionally, due to many beneficial factors (e.g., mild reaction conditions, use of “green” solvents, safer reaction path compared to acid-base neutralization, non-toxic by-product, high atom economy exceeding 90%), this approach can be classified as compatible with principles of green chemistry [40,41].

The synthesized salts are summarized in Table 1. The average yields were in the range of 88–96%. It was observed that increasing the length of the alkyl chain(s) did not affect the reaction yields. The energies of the cation-anion interactions in the obtained ILs (Table S1, ESI) are very similar for all cations **1**–**7** and the energies of the chloride anion interactions with ammonium cations, irrespective of the length of the alkyl substituents.

All salts were obtained as pale yellow liquids at room temperature which solidified after several days (**1**–**5**, **8**) to four weeks (**6**,**7**). This means that the obtained products should be referred to as supercooled liquids. This is a phenomenon commonly observed for quaternary ammonium salts and ILs in particular [42]. Structures of the obtained ILs were confirmed by UV, FT-IR, ^1^H and ^13^C NMR spectroscopy. Spectra (Figures S1–S35, Tables S2 and S3) as well as their thorough analysis (pages S39) are provided in the supplementary data.

According to the data in Table 1, all the synthesized iodosulfuron-methyl-based salts (**1**–**8**) showed melting points ranging from approximately 40 °C (for the salt with two octyl groups (**6**)) to approximately 90 °C (for the salt with octadecyl group (**4**)). Nevertheless, their melting points are below the 100 °C threshold, allowing them to be classified as ILs. Interestingly, the sodium salt of iodosulfuron-methyl melts at approximately 154–157 °C, hence we concluded that in the case of this herbicide the replacement of sodium with a bulky organic ammonium cation can result in a significant reduction of the melting point, even more than 100 °C. It has been established that the homologous series of ILs containing an alkyl longer than C_12_ exhibit phenomenon in which melting point values increase with the elongation of the alkyl chain. This phenomenon is attributed to an increase in van der Waals interactions between the non-polar groups, similar to that of linear alkanes [43,44]. A similar trend was observed for the obtained ILs (**2**–**4**), except that, IL 5 containing the longest docosyl group (C_22_) melted at lower temperature (approximately 62 °C).

### 2.2. Solubility

The solubility results of ILs in 10 representative solvents with different Snyder polarity index are presented in Table 2. All HILs were readily soluble in most polar and non-polar organic solvents. For example, ethyl acetate showed good affinity with almost all synthesized ILs, except ILs containing the longest alkyl chain (C_22_). Hexane, being a less polar solvent, failed to dissolve most of the tested compounds and only 8 demonstrated intermediate solubility. The differences in solubility in the least polar solvents (toluene, hexane) are consistent with available literature data and support the thesis that ionic liquids, due to the presence of a polar ionic bond, are often immiscible with solvents of low polarity index [7,45]. Among the ILs obtained, only one product containing cocotrimethylammonium cation (**1**) showed good solubility in water, while the other salts demonstrated very poor solubility. Thus, in generally, the presence of bulky organic cations containing hydrophobic long alkyls instead of sodium ion affects the solubility of ILs by water molecules and results in reduced hydrophilicity. It should be emphasized that sulfonylureas are known potential contaminants of watercourses [46].

Therefore, the low water solubility of potential new ILs can be considered as highly desirable property that limits their mobility in soil and groundwater. Moreover, the reduced mobility of HILs may favor biodegradation pathway and further limit their contamination potential. Moreover, the good affinity of the tested ILs to most of the selected solvents enables simple and rapid development of the commercial formulations, which is still a major challenge nowadays [21].

**Figure 2:**
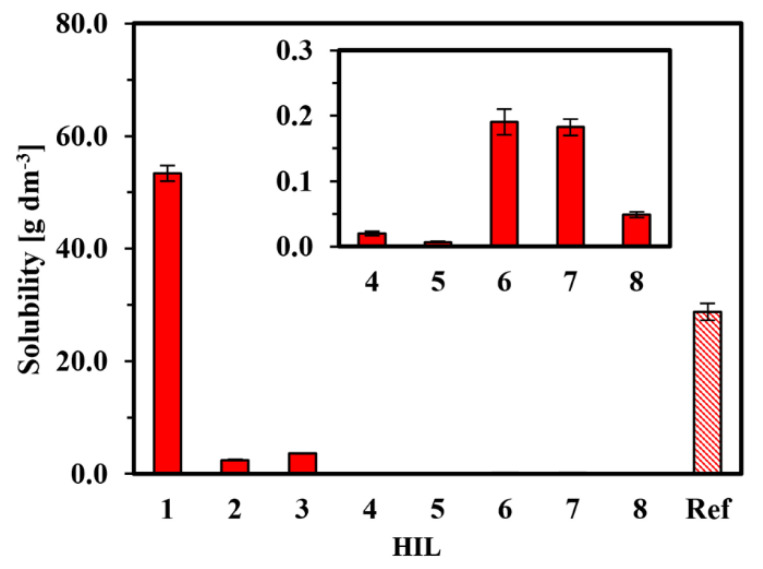
The solubility in the water of the of the obtained ILs (**1**–**8**) in comparison to iodosulfuron-methyl sodium salt (Ref).

Determination of the maximum solubility of the synthesized HILs in water was carried out by spectroscopic method to assess whether the compounds are soluble to a degree suitable for agricultural use. Solubility data is presented in the Figure 2 and in supporting information (Table S3, ESI). Only **1** with the shortest alkyl chain showed a higher affinity for water (approx. 52 g·dm^−3^) compared with sodium salt of iodosulfuron-methyl. HILs **4**–**8** exhibited solubility lower than 0.2 g·dm^−3^ which exceeds the usual application dose of active substance equal to 0.05 g·dm^−3^. In contrast, the solubility of **5** was significantly lower than the recommended application dose, which hinders its effective use as an herbicide. Nevertheless, the following experiment clearly demonstrates that by combining the herbicidal anion with a cation containing the appropriate alkyl chain length this parameter can be easily adjusted.

### 2.3. Octanol-Water Partition Coefficient

**Figure 3:**
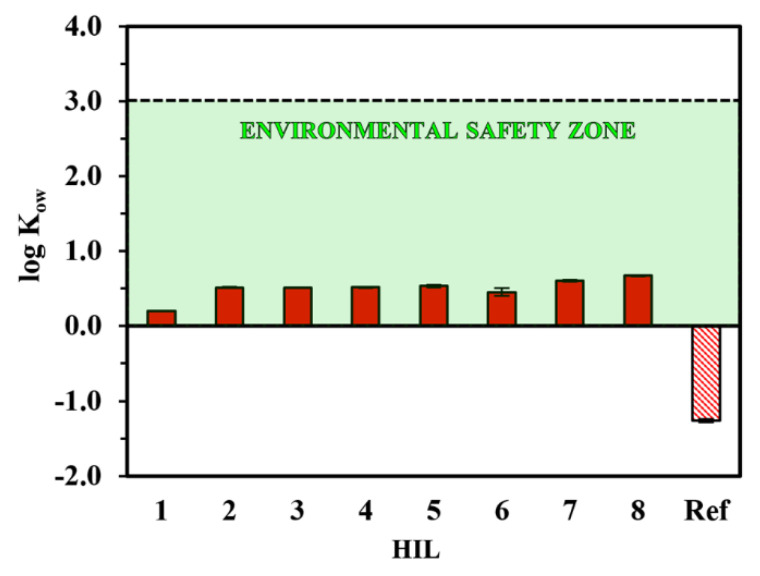
The values of the logarithm of the octanol–water partition coefficient of the obtained ILs (**1**–**8**) in comparison to iodosulfuron-methyl sodium salt (Ref).

The values of octanol-water partition coefficient (log K_OW_) for all the products are shown in Figure 3 (for more data see Table S4, ESI). The results obtained are consistent with the literature data, according to which the replacement of the polar inorganic cation (sodium) with cations comprising at least one long hydrophobic alkyl group leads to an increase in the log K_OW_ values [47,48]. Thus, the iodosulfuron-methyl sodium salt was had the lowest value (log K_OW_ ≈ −1.3), while all products had a log K_OW_ greater than 0 (in the range 0.2–0.7).

The data collected for both types of cations (alkyltrimethylammonium (**1**–**5**) and alkyltrimethylammonium (**6**–**8**)) show a trend that the log K_OW_ value gradually increases with the elongation of the alkyl group of the ILs, which corroborates with other reports [7]. Moreover, the increase in alkyl length from tetradecyl (**2**) to docosyl (**5**) was negligible. It should be emphasized that the log K_OW_ values for all the synthesized ILs ranged from 0 to 3, indicating their superiority over the currently used form, the sodium salt.

This is supported by the fact that, according to literature studies, hydrophilic compounds log K_OW_ values less than zero permeate through soil more easily and pose a threat to groundwater [7]. In contrast, highly lipophilic substances, whose log K_OW_ values are greater than 3, can persist in the soil for many months, which can lead to bioaccumulation [49]. On the data collected, it was hypothesized that the risk of migration of synthesized ILs (**1**–**8**) into groundwater after application is significantly reduced compared to that of the sodium salt. Additionally, the extremely low dose of sulfonylureas (up to 100 times lower compared to other herbicides) combined with optimal log K_OW_ values of ILs creates low probability of their bioaccumulation in soil.

### 2.4. Hydrolysis of Anion in Aqueous Solution

**Figure 4:**
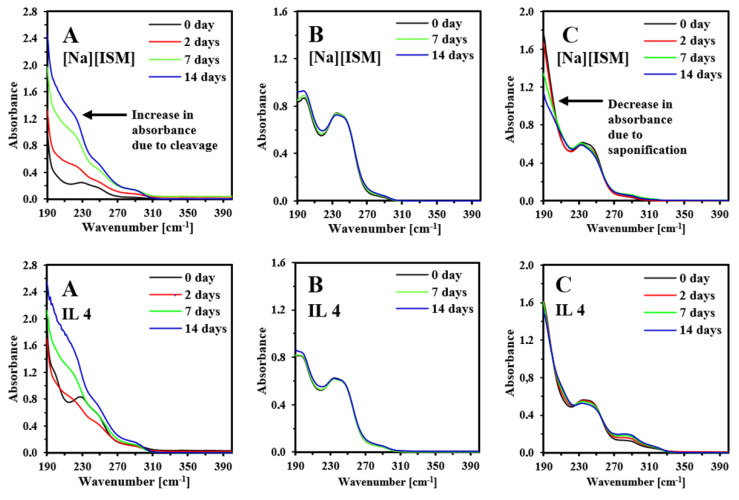
UV spectra of iodosulfuron-methyl sodium salt (**[Na][ISM]**) and IL **4** in acidic (**A**), neutral (**B**) and basic (**C**) environment.

**Scheme 2:**
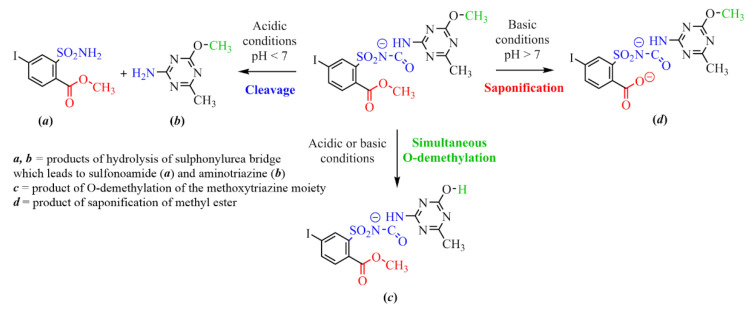
Main routes of hydrolysis of iodosulfuron-methyl anion depending on the pH of the aqueous solution.

Studies on the hydrolysis of sulfonylurea herbicide compounds have been conducted extensively in the past and have provided extremely useful information on their environmental fate [2]. It is known that sulfonylureas are unstable in aqueous media—their hydrolysis half-lives, depending on pH, range from a few days to up to several hundred days. For example, for metsulfuron-methyl and iodosulfuron-methyl the measured half-lives in acidic or neutral soils ranged from 5 to 190 days. Three main metabolic pathways of hydrolysis have been reported for them (see Scheme 2): (1) hydrolysis of the sulfonylurea bridge which leads to formation of sulfonoamide (*a*) and aminotriazine (*b*); (2) O-demethylation of the methoxytriazine moiety (*c*) which can lead to ring opening of the triazine; and (3) saponification of methyl ester (*d*), which appears to be predominant at pH values above 10. The most active ILs (alkyltrimethylammonium (**2**–**4**) and dialkyldimethylammonium (**8**)) were selected to determine their stability in acidic (pH = 3), neutral (distilled water) and basic (pH = 11) aqueous solutions. The same susceptibility to hydrolysis was observed for these ILs, therefore for clarity of presentation only IL **4** is compared with the sodium salt of iodosulfuron-methyl in Figure 4. The results for ILs **2**, **3** and **8** are shown in Figures S36 and S37 (ESI).

The data obtained indicate that the conversion of sulfonylurea to ILs can be favorable and hinder hydrolysis of the anion in alkaline environment, whereas in neutral or acidic conditions the effect of cation structure is negligible. Since the average application of commercial sulfonylureas formulations is recommended to be carried out at pH > 8, our approach may be beneficial to increase their stability [50]. It was observed that both **4** and the sodium salt decompose rapidly in acidic environment (Figure 4A). In the presence of hydronium ions the significant amounts of sulfonylurea precipitated from the solution (82% for sodium salt; 68% for **2**; 77% for **3**; 21% for **4** and 78% for **8**). The structure of the cations affected the precipitation rate of the herbicide, but an increase in absorbance (190–230 nm) suggested decomposition of the anion to water soluble substances. After 2 days most of the sulfonylurea was hydrolyzed, confirming the literature data [2].

Under neutral conditions (Figure 4B) all samples appeared stable. After 14 days only a slight increase in the first maximum at approx. 190 nm (equal to 7% for sodium salt and less than 4% for ILs) and low decrease in the second maximum at approx. 240 nm (in all cases lower than 2%) were observed. According to the literature, alkaline conditions promote saponification of sulfonylurea, which leads to hydrolysis of the ester bond [2]. In Figure 4C a decrease in the absorbance of the first maximum was observed (by approx. 35% for sodium salt within 14 days), which can be attributed to the hydrolysis of the methyl ester. In the spectra of all ILs, the decrease in absorbance was multiple times lower (approx. 5–7% for **2**–**4** and 20% for **8**), leading to the conclusion that the presence of bulky organic cations in such ILs increases their stability in alkaline environment.

It was found that the interaction energy of the ISM anion with the cation in HILs is higher than with the hydroxyl anion, so that the cation is able to block the interaction of the anion at pH >7 (a negative value for ISM—OH equal to −60.02 kcal∙mol^−1^ means no spontaneous attraction). The hydronium cation interacts with the ISM anion more strongly than the ammonium cations (E_w_ = 138.71 kcal∙mol^−1^), and can therefore displace the cation from the ion pair. As a result, the tetraalkylammonium ion does not protect the anion from hydrolysis. For water molecules, the interactions with the anion are weaker than with ammonium cation (ISM—H_2_O E_w_ = 6.52 kcal∙mol^−1^; ISM—3 H_2_O E_w_ = 16.86 kcal∙mol^−1^; ISM—5 H_2_O E_w_ = 27.40 kcal∙mol^−1^), but it should be remembered that there are many water molecules in solution and the total energy of the interactions allows the hydrolysis to occur.

### 2.5. Quantitative Estimation of Emission of Volatile Organic Compounds (VOC)

Volatilization of many currently utilized herbicides after application, usually referred to as “vapor drift”, is a well-known problem that can lead to many negative phenomena, such as the risk of poisoning or the presence of such chemicals in neighboring areas which can cause damage to non-targeted plants as well as trees. Therefore, all new herbicides should be tested for their potential to emit volatile organic compounds (VOCs) to ensure that they do not pose a threat. In this study the emissions of the volatiles from all synthesized HILs were determined. In the experiment, the exact mass of the compounds introduced into the vial, set as 0.2 g for each compound, allowed the calculation of the percentage of volatile compounds released from the sample.

The estimation of the mass of the VOCs were made on the basis of the area of chromatographic peaks obtained for injected vapors of each compound. The results are presented in Table S6, ESI. The emissions of VOC at 35 °C were recorded for almost all compounds, tested except for HILs **2**, **4** and sodium salt of iodosulfuron-methyl. In general, most of the products obtained emitted very small amounts of VOCs (approx. 0.02–0.06%). Compound **7** showed the highest VOC emission, but it was less than 1 %. It should be noted that the iodosulfuron-methyl anion has functional groups known to be susceptible to degradation, in particular the sulfonylurea bridge, ester and ether bond. According to recent report,^7^ the iodosulfuron-methyl, when stored at elevated temperature (75 °C), decomposed entirely just after a few days of the experiment. Therefore, it might be expected as the emitted compounds are the effect of the degradation of the tested compounds, however, further studies are needed to prove this hypothesis.

### 2.6. Herbicidal Activity

**Figure 5:**
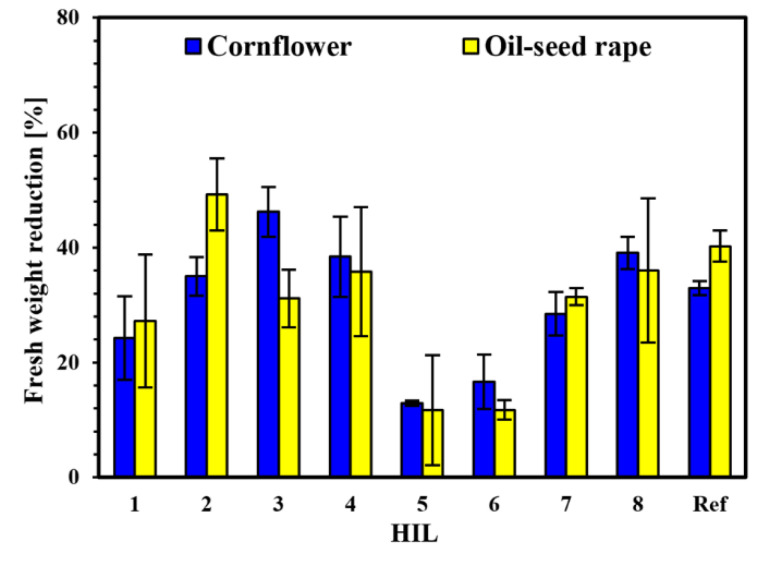
Herbicidal activity of the synthesized HILs (**1**–**8**) compared to commercial preparation Huzar 05 WG (Ref) toward cornflower and oil-seed rape.

The herbicidal activity of the synthesized HILs was tested against two representative weeds: cornflower and oil-seed rape. The commercial formulation of the reference herbicide was Huzar 05 WG containing the sodium salt of iodosulfuron-methyl. It also contains heavy aromatic hydrocarbons and unspecified petroleum fraction, which are responsible for its high biological activity. The herbicidal activity of the obtained HILs is shown as reduction in fresh weight of weeds in Figure 5 (see Table S7, ESI for more data). HIL with myristyltrimethylammonium cation (**2**) showed the highest efficacy and was more active than Huzar 05 WG alone against both plants (Figure 5). On the other hand, HIL 3 showed the highest activity against cornflower among all the tested compounds, and HIL **5** with the longest chain (C_22_) showed the lowest herbicidal activity. This was due to its increased hydrophobicity, which limited its solubility in water below the application dose. Both ILs **7** and **8** were equally effective as reference substances.

**Figure 6:**
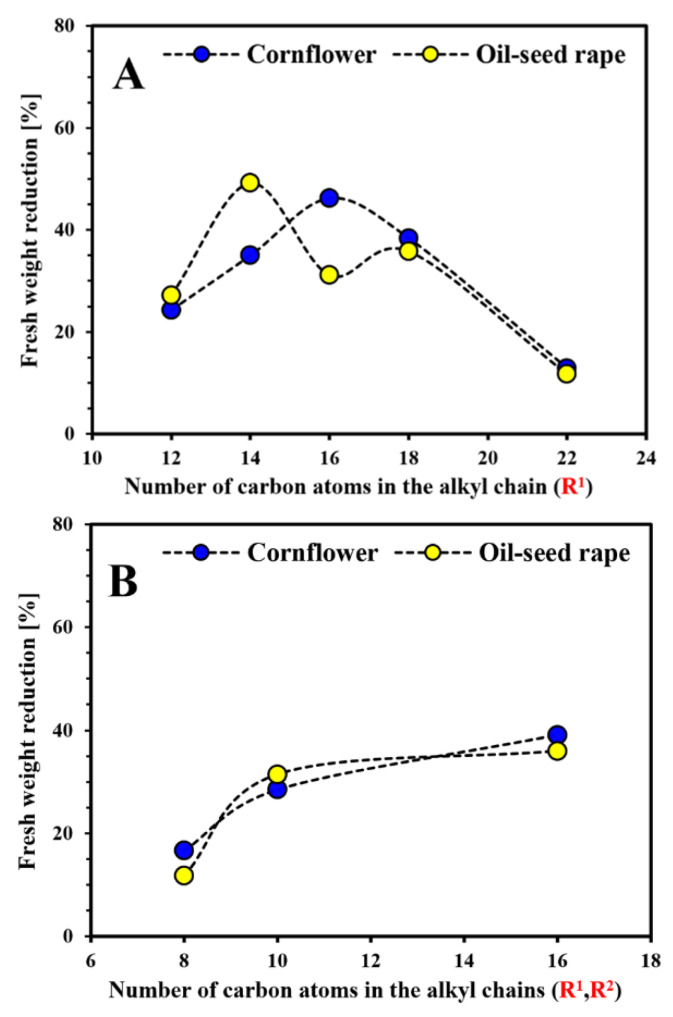
Influence of the alkyl chain length of the obtained HILs **1**–**5** (**A**) and **6**–**8** (**B**) on the effectiveness of fresh weight reduction of weeds. R^1^, R^2^—long alkyl chain.

The optimum alkyl chain length present in the alkyltrimethylammonium cation was 14–16 carbon atoms for winter oilseed rape and cornflower, respectively (Figure 6A). These data are consistent with available reports [29,44]. For cations containing two long alkyl chains, high activity was observed for at least 10 carbon atoms.

The synthesized products retained the biological activity of the herbicide iodosulfuron-methyl and can be classified as new herbicidal ionic liquids. It should be emphasized that most of the obtained HILs show efficacy comparable to the commercial formulation without adjuvant addition. HILs allow the elimination of the use of other chemicals, which is significantly beneficial in terms of their potential environmental impact. Therefore, these compounds can be considered as potential replacements for currently used sulfonylurea-based herbicides.

### 2.7. Determination of Antimicrobial Activity

Toxicity studies were conducted to assess the possible environmental impact of HILs. Two microorganisms isolated from specific environmental niches were analysed: Gram-negative *P. putida* and Gram-positive *B. cereus*. The toxicity results, presented as EC_50_ values in Table 3, obtained for the iodosulfuron-methyl-based salts were significantly different for *P. putida* and *B. cereus*. Except for **2**, **6** and **7**, all the substances were practically harmless to *P. putida* and showed toxicity above 200 mg·dm^−3^ (identical to the reference—Iodosulfuron-methyl sodium salt).

This is consistent with the results reported in material safety data sheets [24], where for sodium salt of iodosulfuron-methyl the EC_50_ after 72 h for *P. putida* was above 1000 mg·dm^−3^. In contrast, all products were slightly toxic (<50 mg·dm^−3^) to *B. cereus*. A similar effect was not observed for the sodium salt. The observed differences in ecotoxicity of HILs against Gram-positive and Gram-negative have already been reported in our previous study [51] and may be due to different defence mechanisms against the tested substances.

### 2.8. Mobility in Soil

It should be noted that many sulfonylureas herbicides exhibit high mobility in soil, thus posing a real risk of leaching to groundwater, as demosntrated in many studies [2,52,53]. For example, other sulfonylurea known as nicosulfuron and its metabolites have been detected in surface water as well as groundwater around the world [54,55]. As shown in Table S8, ESI, iodosulfuron−methyl sodium salt and HILs **1**, **2**, **6**–**8** exhibited high mobility in soil (Rf = 0.93–0.98). In contrast, **3**–**5** were uniformly distributed along the length of the TLC plate. The likely explanation for this behavior could be the increased hydrophobicity of the obtained compounds. This suggests that the transformation of iodosulfuron-methyl to the IL form may slow down the mobility of the herbicide in the soil profile.

## 3. Materials and Methods

### 3.1. Materials

Myristyltrimethylammonium bromide 99% from Sigma-Aldrich, Saint Louis, MO, USA. Stearyltrimethylammonium chloride (80% isopropanolic solution) and behenyltrimethylammonium chloride (80% isopropanolic solution) were purchased from KCI Limited, Chungnam, South Korea. Arquad 2HT (di(hydrogenated tallow)dimethylammonium chloride, 75% isopropanolic solution), Arquad C-35 (cocotrimethylammonium chloride, 35% aqueous solution) and didecyldimethylammonium chloride (40% aqueous solution) were obtained from Akzo-Nobel, Amsterdam, The Netherlands. Dioctyldimethylammonium chloride (99%) was purchased from CHEMOS GmbH, Altdorf, Germany. Cetyltrimethylammonium chloride (50% aqueous solution) was obtained from Stockmeier Chemie, Poznan, Poland. Iodosulfuron-methyl sodium salt (97%) was from Pestinova, Jaworzno, Poland. All solvents (methanol, DMSO, acetonitrile, acetone, isopropanol, ethyl acetate, chloroform, toluene, hexane) and potassium hydroxide were obtained from Avantor, Gliwice, Poland. Deionized water with conductivity <0.1 µS·cm^−1^, was obtained from demineralizer Hydrolab HLP Smart 1000, Straszyn, Poland. All tetraalkylammonium halides were thoroughly dried at 50 °C for 48 h under reduced pressure (1–2 mbar) prior to the syntheses.

### 3.2. Syntheses

#### 3.2.1. Classical Method of Synthesis of ILs

The appropriate tetraalkylammonium halide (0.01 mol) was dissolved in 15 cm^3^ of water in a 100 cm^3^ reaction vessel equipped with a mechanical stirrer. Then, a stoichiometric amount (0.01 mol) of the sodium salt of iodosulfuron-methyl dissolved in 15 cm^3^ of water was added to the reactor for ion exchange reaction. The products precipitated from the aqueous solution. A two-phase extraction technique was used to isolate the products from the reaction mixture. For this purpose, 30 cm^3^ of chloroform was added to the reaction mixture and both phases were mixed thoroughly. The organic layer was then separated and washed 5 times with 20 cm^3^ of distilled water. The presence of halides in the organic phase was controlled by adding a few drops of the 0.1 M AgNO_3_ solution to each sample of aqueous phase after separation. About 3 g of anhydrous Na_2_SO_4_ was added to remove the traces of water from the organic phase. After 5 minutes, the Na_2_SO_4_ was filtered and the solvent was evaporated from the filtrate. Finally, the obtained products were dried at 40 °C for 24 h under reduced pressure (1–2 mbar). All the synthesized salts were stored in a desiccator over a drying agent (P_4_O_10_).

#### 3.2.2. Simplified Method of Synthesis of ILs

In a reaction flask, 0.03 mol of stearyltrimethylammonium chloride or di(hydrogenated tallow)dimethylammonium chloride and 15 cm^3^ of methanol/ethanol were mixed. Then, 15 cm^3^ of the methanolic/ethanolic solution containing stoichiometric amounts of the iodosulfuron-methyl sodium salt was slowly added. The reaction was carried out under constant stirring at 40 °C for 15 min. The by-product (sodium chloride) precipitated from the reaction solution. The inorganic salt was then separated by filtration, and alcohol was evaporated from the filtrate. The crude product was dried and then dissolved in 30 cm^3^ of anhydrous acetone. The precipitate formed was separated by vacuum filtration and the solvent was evaporated. The amount of precipitate in a filter from the second filtration was accurately weighed to determine the NaCl content of each crude product. The reaction yields for all tests performed were in the range of 91–96%.

### 3.3. Methods

#### 3.3.1. Spectral Analysis

^1^H NMR spectra were recorded on a Mercury Gemini 300 (Varian, Inc., Palo Alto, CA, USA) operating at 300 MHz and on a Varian VNMR-S 400 MHz (Varian, Inc., Palo Alto, CA, USA) with TMS as internal standard. ^13^C NMR spectra were collected using the same instruments at 75 MHz and 100 MHz. IR spectra (3000 to 650 cm^−1^ with 8 cm^−1^ resolution) were collected using semi-automated system EasyMax 102 (Mettler Toledo, Greifensee, Switzerland) coupled to a ReactIR iC15 probe equipped with an MCT detector and a 9.5-mm AgX diamond tipped probe and processed using iCIR 4.3 software. UV absorption studies were performed for each of the obtained compounds on a Rayleigh UV-1601 apparatus (BRAIC, Beijing, China). Methanolic solutions of the synthetized products (at a concentration of approx. 0.05 mg·cm^−3^) were used for determine the molar absorption coefficient. Pure methanol was used as a reference standard. Spectra were obtained in the wavelength range λ = 190–400 nm in water. In experiments to determine stability factors and octanol-water partition coefficients, water or octanol saturated water was used as reference.

#### 3.3.2. Melting Point

Melting points of compounds were determined using Mettler Toledo MP 90 melting point determination system (Mettler Toledo, Greifensee, Switzerland). The temperature gradient was equal to 10 °C·min^−1^.

#### 3.3.3. Solubility

##### Method 1

The solubility of the obtained ILs was analyzed in ten representative solvents according to the protocols in Vogel’s Textbook of Practical Organic Chemistry [56]. The solvents were arranged in descending order of Snyder’s polarity index: water—9.0, methanol—6.6, DMSO—6.5, acetonitrile—6.2, acetone—5.1, ethyl acetate—4.3, isopropanol—4.3, chloroform—4.1, toluene—2.3, and hexane—0.0. A 0.1 g sample of each IL was added to a defined volume of solvent and the samples were then thermostated in a Memmert Model WNB 7 water bath (Memmert GmbH, Büchenbach, Germany) at 25 °C. Depending on the volume of solvent used, 3 types of behavior were recorded: ‘soluble’ for compounds that dissolved in 1 cm^3^ of solvent (>10%), ‘limited solubility’—For compounds that dissolved in 3 cm^3^ of solvent (3.3–10%), and ‘not soluble’—For compounds that did not dissolve in 3 cm^3^ of solvent (<3.3%).

##### Method 2

The exact solubility of 1–8 and iodosulfuron-methyl sodium salt in water was evaluated according to OECD guidelines [57]. 0.1 g of the substance was placed in a vial and mixed with water for 24 h, 48 h and 72 h. The samples were then centrifuged and the liquid phase were collected using a syringe. The concentrations of the compounds in water were determined spectrophotometrically using a UV/Vis spectrophotometer (based on formerly made calibration curves with plots of absorbance (at λ_max_ = 235 nm in water) vs. concentration for each substance). Three replicates of each measurement were performed.

#### 3.3.4. Octanol-Water Partition Coefficients

Octanol-water partition coefficients (K_OW_) of the synthesized ionic liquids (**1**–**8**) and sodium salt of iodosulfuron-methyl were evaluated using the shake-flask method according to OECD guidelines [58]. K_OW_ values were measured using mutually saturated distilled water and *n*-octanol in a glass vial with a magnetic stir bar. First, iodosulfuron-methyl sodium salt and synthesized products were dissolved in 5 cm^3^ of distilled water in amounts used in greenhouse experiments (similar to those used in agriculture by farmers—7.5 g of active ingredient per hectare), and then 5 cm^3^ of octanol was added. All vials were shaken at 25 °C. After 24 h, the samples were centrifuged and the aqueous and octanol phases were collected using a syringe. The concentrations of compounds in water were determined spectrophotometrically using a UV/Vis spectrophotometer (based on formerly made calibration curves with plots of absorbance (at λ_max_ = 235 nm in water) vs. concentration for each substance). Three replicates of each measurement were performed.

#### 3.3.5. Hydrolysis in Water

Hydrolysis of selected ILs (**2**–**4** and **8**) and iodosulfuron-methyl sodium salt was evaluated for aqueous solutions at a concentration of 74.07 µmol·dm^−3^ (corresponding to a dose of 7.5 g of herbicidal anion per hectare). Measurements were made in 0.5 dm^3^ volumetric flasks with a magnetic stirring bar. An appropriate amount of IL or sodium salt was introduced into the flask and dissolved in 500 cm^3^ of 0.001 M hydrochloric acid solution (pH = 3) (*a*), distilled water (*b*), or 0.001 M sodium hydroxide solution (pH = 11) (*c*). All flasks were shaken in a Heidolph MR Hei- stirrer End (Heidolph, Schwabach, Germany) equipped with an anodized Heat-On block at a constant temperature of 25.0 °C (with accuracy ± 0.1 °C) in the dark. After a specific period of time, a 1 cm^3^ sample was taken from each flask (solutions of compounds in distilled water and sodium hydroxide were diluted 3-fold). The absorbance of each sample (190–400 nm) was measured using a Rayleigh UV-1800 spectrophotometer (BRAIC, Beijing, China). The concentrations of the precipitated herbicide from hydrochloric acid were determined from calibration curves using plots of absorbance as a function of concentration for each substance.

#### 3.3.6. Structure Modeling

Structural modeling was carried out in HyperChem 8.0.6 software. Individual models were built using a graphical interface and then optimized by the semi-empirical PM3 method using the Fletcher-Reeves algorithm (conjugate gradient) on isolated chemical individuals (i.e., in a vacuum). Calculations were carried out until the total energy gradient was less than or equal to 0.01 kcal·mol^−1^. The interaction energy between the components of the complex was calculated as a number opposite to the standard enthalpy of the complex formation reaction, as the difference between the standard heats of formation of the complex and its components:

(1) $$E_{w}=-\Delta H_{r}=-(HoF_{complex}-\sum HoF_{individuals})$$

#### 3.3.7. Quantitative Estimation of Emission of Volatile Organic Compounds (VOC)

A gas chromatograph (Clarus580, PerkinElmer, Waltham, MA, USA) equipped with an FID and coupled to a head-space injector (TurboMatrix HS 40, PerkinElmer, Waltham, MA, USA) was used to quantify the amount of volatile organic compounds emitted by the tested compounds. Emissions were studied at 35 °C. 0.2 g of each studied compounds were placed separately in 20 mL vials and sealed. They were placed in an autosampler. The vials were then thermostated at 35 °C for 20 min. After this time the entire volume of the vials was collected by an automatic valve and injected into the chromatographic column. GC analysis was performed at 200 °C. Quantitative analysis was performed using inner standard procedure, injecting a known a known amount of the tested compounds to estimate the detector response factor.

#### 3.3.8. Herbicidal Activity

Seeds of rapeseed (*Brassica napus* L.) and cornflower (*Centaurea cyanus* L.) plants were used to study the biological activity of the tested compounds. Seeds of selected plants were sown into plastic pots (1.0 dm^3^, 15 cm diameter) containing a peat-based substrate. Plants were grown in a greenhouse with a photoperiod of 16 h day and 8 h night. Temperature was maintained at 25 ± 2 °C during the day and 20 ± 2 °C at night. Relative humidity was set at 60%. Seedlings were reduced to four uniform plants per pot for rapeseed and to five uniform plants per pot for cornflower. Trials were designed as a randomized complete block with four replications. The reference herbicide and synthetized compounds were applied at the 4 leaf stage of the plants (BBCH 14) using a laboratory sprayer equipped with a spray chamber using Tee Jet 1102 (TeeJet Technologies GmbH, Schorndorf, Germany) nozzles at a rate of 200 dm^3^·ha^−1^ at 0.2 MPa. ILs (**1**–**8**) were dissolved in water to a concentration corresponding to a dose of 7.5 g of active ingredient (anion) per hectare. This corresponds to the recommended dose used by farmers. A commercial product containing the iodosulfuron-methyl sodium salt (Huzar 05 WG, Bayer CropScience, Leverkusen, Germany) was applied at the same dose of active ingredient. The sprayer was moved at a constant speed of 3.1 m·s^−1^ at a height of 40 cm above the plants. After treatment, plants were again placed in the greenhouse under established environmental conditions. Two weeks after treatment, plants were cut at soil level and weighed with 0.01 g accuracy. The results of the experiment were expressed as percent of the fresh weight reduction in comparison to the weight of control objects (plants untreated with any herbicidal formulations). Each error interval range represents standard errors of the mean (SEM). The SEM values were calculated according to the following equation:

(2) $$SEM=\frac{s}{n^{0.5}}$$

where:

*SEM*—standard error of the mean

*s*—sample standard deviation

*n*—number of samples.

#### 3.3.9. Determination of Soil Mobility

The mobility of HILs in soil was investigated using a soil thin layer chromatograph (soil TLC) according to the procedure described by Tang et al. [32]. The collected agricultural soil was air-dried, and sieved through a 2-mm diameter sieve and then fed into an electric mill. The resulting powder was sieved through a 100 µm mesh sieve. 10 g of the powdered soil was suspended in water to obtain a slurry. The obtained suspension was applied to a 10 × 13 cm glass plate using a brush and the thickness of the soil layer was 0.7 mm. The plates were dried at room temperature for 24 h after obtaining an even distribution of the suspension. Two lines were plotted on each plate at distances of 1.5 cm and 11.5 cm above the soil. The test compounds in acetone were dropped with a microsyringe at a distance of 1.5 cm from the bottom edge of the plate. The plates were immersed in water as eluents in a closed chromatographic chamber at a height of 0.5 cm below the start line. The plates were removed when the eluent moved to the 11.5 cm line and dried at room temperature. Rf values were determined from the furthest distance traveled by the test compounds divided by the distance traveled by the eluent front. The experiments were performed in triplicate.

#### 3.3.10. Antimicrobials Activity Testing

Stock solutions (10 g·dm^−3^) of salts based on iodosulfuron-methyl as reference were diluted in acetone. Further concentrations of 1000, 750, 500, 375 and 250 mg·dm^−3^ were prepared by diluting the stock solutions in sterile TSB medium (50%, *v/v*, Sigma Aldrich, Saint Louis, MO, USA). All solutions were stored at 4 °C until use, but no longer than 5 days. Two bacterial strains, *P. putida* (Gram-negative) and *B. cereus* (Gram-positive) were used to evaluate the antimicrobial activity of the tested substances. Each culture was transferred from agar plates to 50% TSB broth with an optical density (OD_600_) of approx. 0.100 ± 0.015. Cultures (0.2 cm^3^) were incubated in sterile 96-well plates in triplicates at 30 °C with continuous shaking in a Synergy™ HTX Multi-Mode Microplate Reader until the bacterial suspension reached an OD_600_ of 0.150 ± 0.015. Then, 0.05 cm^3^ of each of the previously prepared solutions were added to the wells to obtain the final test concentration of 200, 150, 100, 75 and 50 mg·dm^−3^. The bacterial strains were further cultivated under the above conditions for 16 h. Abiotic (bacterial medium without microorganisms and chemical compounds), biotic (bacterial medium with microorganisms) and bacterial medium without microorganisms but with the addition of analyzed compounds were used as controls. All substances and controls were prepared in triplicate. After the bacterial strains reached stationary phase, growth curves were plotted and then the rate of inhibition of microbial growth was calculated. The half maximal effective concentration (EC50·t^−1^) was determined according to the procedure described by Syguda et al. [59].

## Figures and Tables

**Table 1:** Synthesized iodosulfuron-methyl-based salts with commercial tetraalkylammonium cations (**1**–**8**).

No.	Structure of Cation	R ^1^	R ^2^	Yield [%]	Melting Point [°C]
1	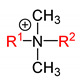	*coco* ^1^	CH_3_	88	67.0–69.5
2		C_14_H_29_	CH_3_	92	64.3–66.1
3		C_16_H_33_	CH_3_	91	76.2–78.5
4		C_18_H_37_	CH_3_	96	89.3–91.1
5		C_22_H_45_	CH_3_	92	61.0–62.6
6		C_8_H_17_	C_8_H_17_	85	40.4–42.5
7		C_10_H_21_	C_10_H_21_	92	51.1–52.2
8		*hydrogenated tallow* ^2^	*hydrogenated tallow*	95	49.0–52.9

^1^ *coco*: mixture of saturated alkyl substituents C_8_—5%, C_10_—6%, C_12_—50%, C_14_—19%, C_16_—14%, C_18_—10%; ^2^ *hydrogenated tallow*: mixture of saturated (97%) or unsaturated (3%) alkyl substituents. C12—1%; C14—4%; C16—31%; C18—64%.

**Table 2:** Solubility of the prepared ILs (**1**–**8**) at 25 °C.

Salt	Water	Methanol	DMSO	Acetonitrile	Acetone	Chloroform	Isopropanol	Ethyl Acetate	Toluene	Hexane
	9.0 ^a^	6.6	6.5	6.2	5.4	4.4	4.3	4.3	2.3	0.0
1	+	+	+	+	+	+	+	+	+	–
2	−	+	+	+	+	+	+	+	+	−
3	−	+	+	+	+	+	+	+	±	−
4	−	+	+	+	+	+	+	+	+	−
5	−	+	+	+	+	+	+	±	+	−
6	−	+	+	+	+	+	+	+	±	−
7	−	+	+	+	+	+	+	+	±	–
8	−	+	+	+	+	+	+	+	+	±
REF ^b^	±	+	+	±	±	−	−	−	−	−

^a^ Snyder polarity index; ^b^ iodosulfuron-methyl sodium salt; “+” good solubility; “±” medium solubility; “−” low solubility.

**Table 3:** Half-Maximal Effective Concentrations (EC_50_) of the iodosulfuron-methyl-based salts.

		EC50 ± Standard Error of the Mean [mg·dm^−3^]		
Salt	*P. putida*	Toxicity Classification	*B. cereus*	Toxicity Classification
1	186.06 ± 3.09	PH ^b^	<50	ST
2	<50	ST	<50	ST
3	>200	PH	<50	ST
4	>200	PH	<50	ST
5	>200	PH	<50	ST
6	<50	ST ^c^	<50	ST
7	<50	ST	<50	ST
8	186.32 ± 4.69	PH	53.54 ± 0.46	ST
REF ^a^	>200	PH	>200	PH

^a^ iodosulfuron-methyl sodium salt; ^b^ PH—1000 mg·dm^−3^ practically harmless; ^c^ ST—10–100 mg·dm^−3^ slightly toxic.

## Data Availability

Data are available on request.

## Supplementary Materials

The following are available online, Figure S1/S5/S9/S13/S17/S21/S25/S29. UV spectra of synthesized compounds (**1**–**8**). Figure S2/S6/S10/S14/S18/S22/S26/S30. ^1^H NMR spectra of synthesized compounds (**1**–**8**). Figure S3/S7/S11/S15/S19/S23/S27/S31. ^13^C NMR spectra of synthesized compounds (**1**–**8**). Figure S4/S8/S12/S16/S20/S24/S28/S32. FT-IR spectra of synthesized compounds (**1**–**8**). Figure S33. The FT-IR spectra of product with mirystyltrimethylammonium cation (**2**) with numbering of the most characteristic vibrations Figure S34. The comparison between FT-IR spectra of ILs comprising alkyltrimethylammonium cation (1–5) compared to iodosulfuron-methyl sodium salt ([Na][ISM]). Figure S35. The comparison between FT-IR spectra of products with dialkyldimethylammonium cation (6–8) compared to iodosulfuron-methyl sodium salt ([Na][ISM]). Table S1. The interaction energies of the cation with the anion in the substrates and obtained ILs. Table S2. Analysis of FT-IR spectrum of the product with mirystyltrimethylammonium cation (**2**) Table S3. Analysis of FT-IR spectra of all products compared to sodium salt of iodosulfuron-methyl ([Na][ISM]). Spectral analysis of FT-IR and NMR spectra with references. Table S4. Values of water solubility for ILs 1–8 and sodium salt of iodosulfuron-methyl [Na][ISM] at 25 °C Table S5. Values of logarithm of octanol-water partition coefficient for ILs 1–8 and sodium salt of iodosulfuron-methyl [Na][ISM] at 25 °C. Figure S36. UV spectra of iodosulfuron-methyl sodium salt ([Na][ISM]) and ILs 2–4 and 8 in acidic environment. Figure S37. UV spectra of iodosulfuron-methyl sodium salt ([Na][ISM]) and ILs 2–4 and 8 in basic environment. Table S6. Volatility of the studied compounds at 35 °C. Table S7. Efficacy of the prepared ILs (1–8) toward cornflower (*Centaurea cyanus* L.) and oil-seed rape (*Brassica napus* L.) Table S8. Migration of HILs in agricultural soil (soil TLC analysis).

## Sample Availability

Samples of the compounds are available from the authors.
