# Supplementary material for: Transformation of Iodosulfuron-Methyl into Ionic Liquids Enables Elimination of Additional Surfactants in Commercial Formulations of Sulfonylureas
Source: Molecules. 2021 Jul 21;26(15):4396. doi: 10.3390/molecules26154396 (PMC8348827; doi:10.3390/molecules26154396)
Supplement: Supplementary file 1 [file molecules-26-04396-s001.zip › molecules-1291346-supplementary.pdf]

ELECTRONIC SUPPORTING INFORMATION

Transformation of iodosulfuron-methyl into ionic liquids enables elimination of additional surfactants in commercial formulations of sulfonylureas

Witold Stachowiak [1], Radosław Szumski [1], Jan Homa [1], Marta Woźniak-Karczewska [1], Anna Parus [1], Beata Strzemiecka [1], Lukasz Chrzanowski [1,*] and Michał Niemczak [1]

[1] Department of Chemical Technology, Poznan University of Technology, Poznan 60-965, Poland; witold.stachowiak@doctorate.put.poznan.pl (W.S.); r.szumski@isofcik.eu (R.S.); jan.j.homa@doctorate.put.poznan.pl (J.H.); marta.wozniak-karczewska@put.poznan.pl (M.W.-K.); an-na.parus@put.poznan.pl (A.P.); beata.strzemiecka@put.poznan.pl (B.S.); michal.niemczak@put.poznan.pl (M.N.)

[*] Correspondence: lukasz.chrzanowski@put.poznan.pl

The following abbreviations were used in ultraviolet (UV) spectra:

λ_{max} = the wavelength of maximum absorbance

$\varepsilon_{\lambda max}$ = molar absorption coefficient at λ_{max}

The following abbreviations were used to explain the multiplicities in nuclear magnetic resonance (NMR) spectra:

s = singlet, d = doublet, t = triplet, m = multiplet,

Figure S1*. UV spectrum of cocotrimethylammonium ((5-iodo-2-(methoxycarbonyl)phenyl)sulfonyl)((4-methoxy-6-methyl-1,3,5-triazin-2-yl)carbamoyl)amidate (****1****).*

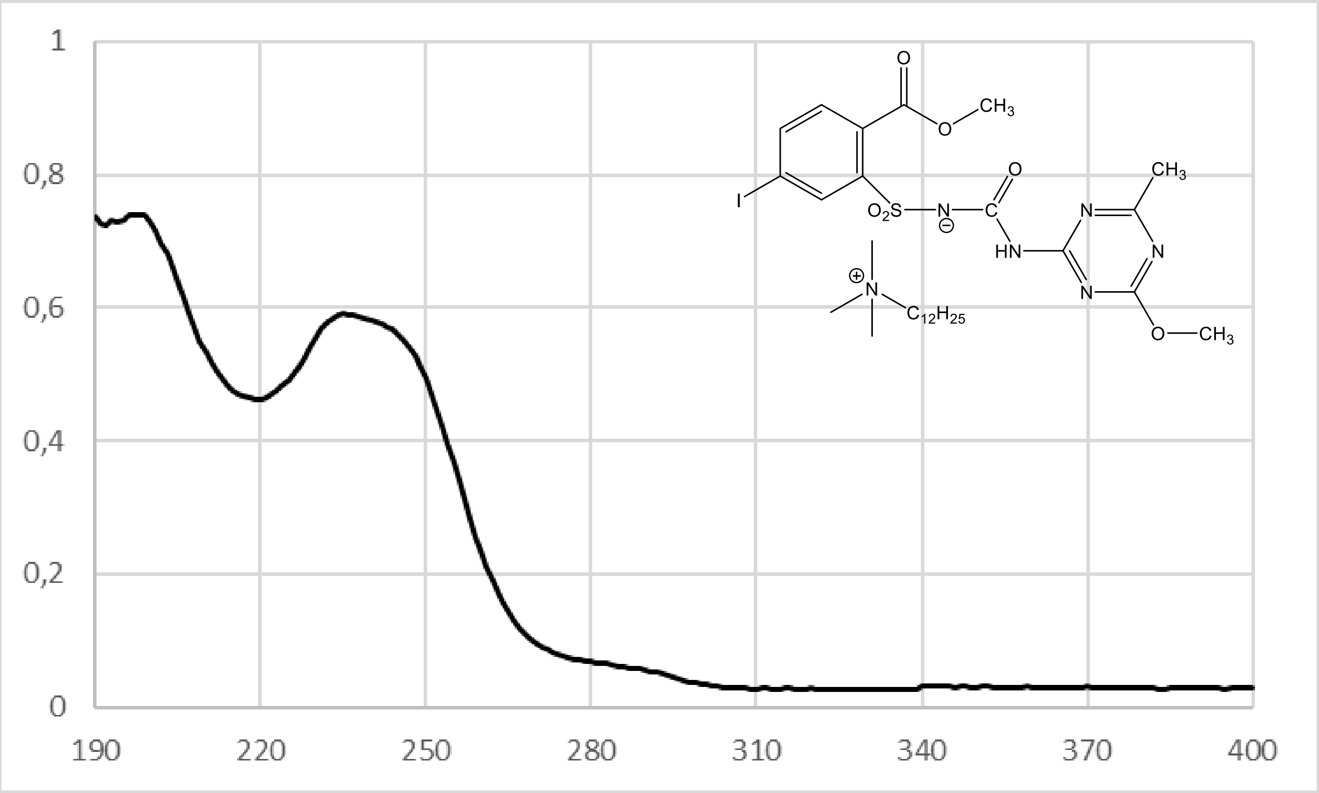

λ_{1max} *(water) = 197 nm;* λ_{2max} *(water) = 235 nm*

$\varepsilon_{\lambda 1max} = 2.93 \cdot 10^4\ dm^3\ mol^{-1}\ cm^{-1}$; $\varepsilon_{\lambda 2max} = 2.34 \cdot 10^4\ dm^3\ mol^{-1}\ cm^{-1}$

$\varepsilon_{\lambda 1max} / \varepsilon_{\lambda 2max} = 1.25$

Figure S2. *FT-IR spectrum of cocotrimethylammonium ((5-iodo-2-(methoxycarbonyl)phenyl)sulfonyl)((4-methoxy-6-methyl-1,3,5-triazin-2-yl)carbamoyl)amidate (**1**).*

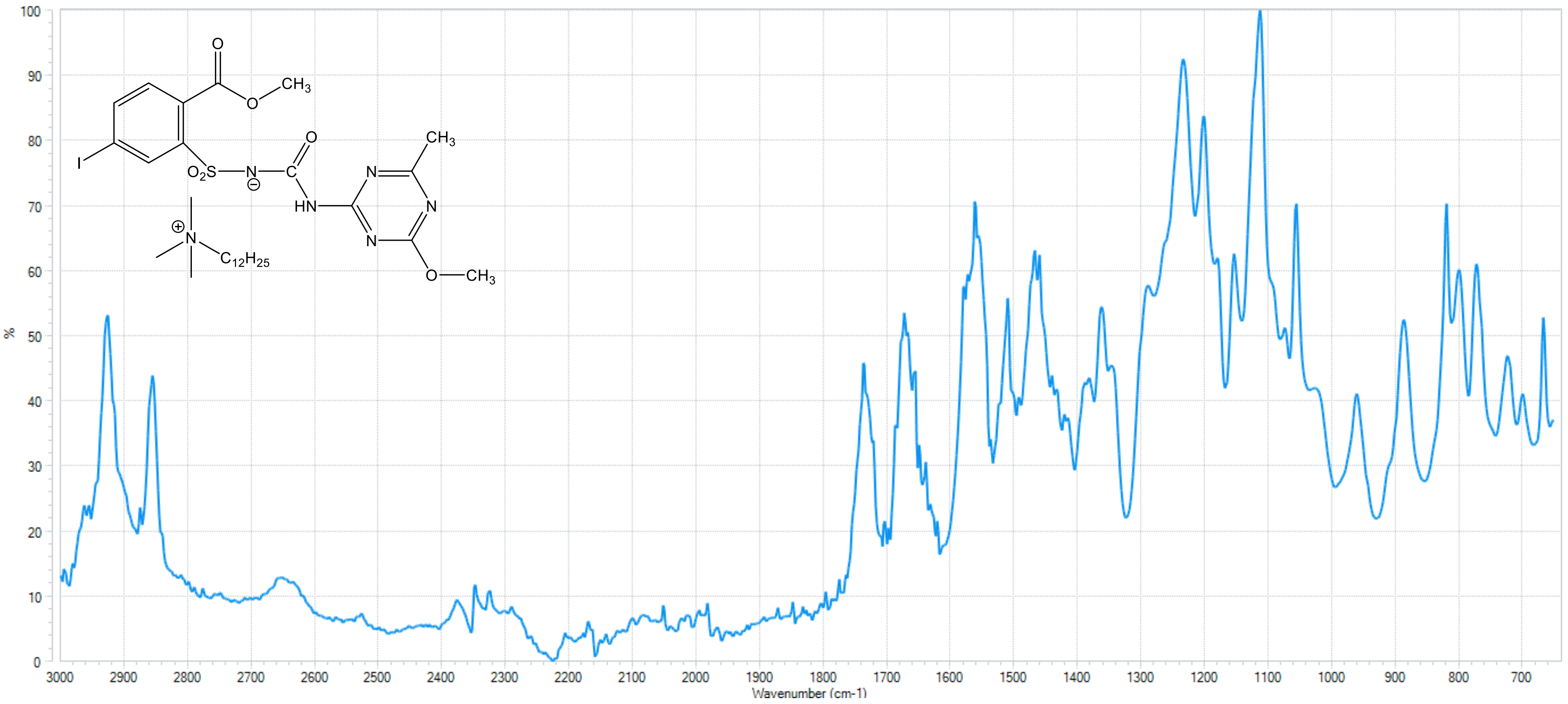

IR υ_{max} [cm^{-1}] = 2930, 2855, 1738, 1672, 1656, 1563, 1511, 1468, 1422, 1361, 1343, 1284, 1232, 1200, 1110, 1055, 888, 819, 771

Figure S3*. ^{1}H NMR spectrum of cocotrimethylammonium ((5-iodo-2-(methoxycarbonyl)phenyl)sulfonyl)((4-methoxy-6-methyl-1,3,5-triazin-2-yl)carbamoyl)amidate (****1****).*

^{1}H NMR (400 MHz, $CDCl_3$) δ [ppm] = 0.88 (t, *J* = 7,0 Hz, 3H), 1.25 (m, 18H), 1.64 (m, 2H), 2.40 (s, 3H), 3.19 (s, 9H), 3.29 (m, 2H), 3.86 (s, 3H), 3.91 (s, 3H), 7.11 (d, *J* = 8.1 Hz, 1H), 7.74 (m, 2H), 8.40 (d, *J* = 1.6 Hz, 1H).

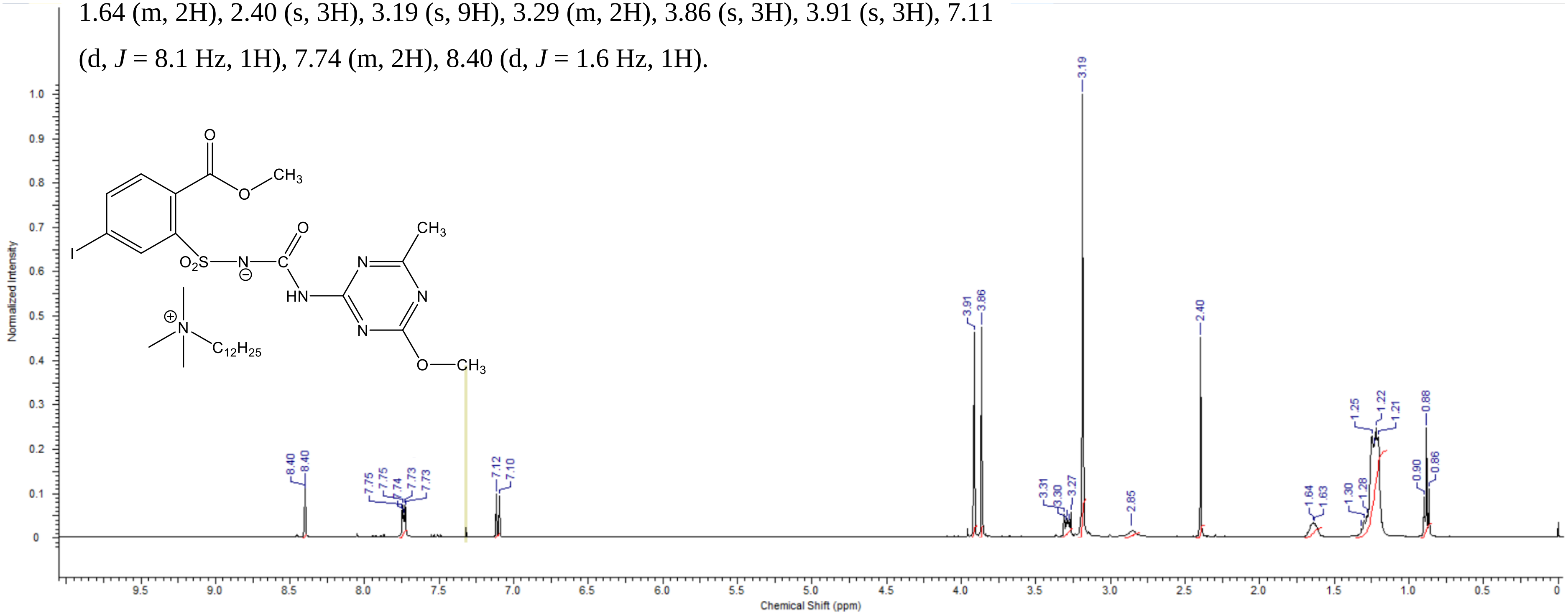

Figure S4. *^{13}C NMR spectrum of cocotrimethylammonium ((5-iodo-2-(methoxycarbonyl)phenyl)sulfonyl)((4-methoxy-6-methyl-1,3,5-triazin-2-yl)carbamoyl)amidate (**1**).*

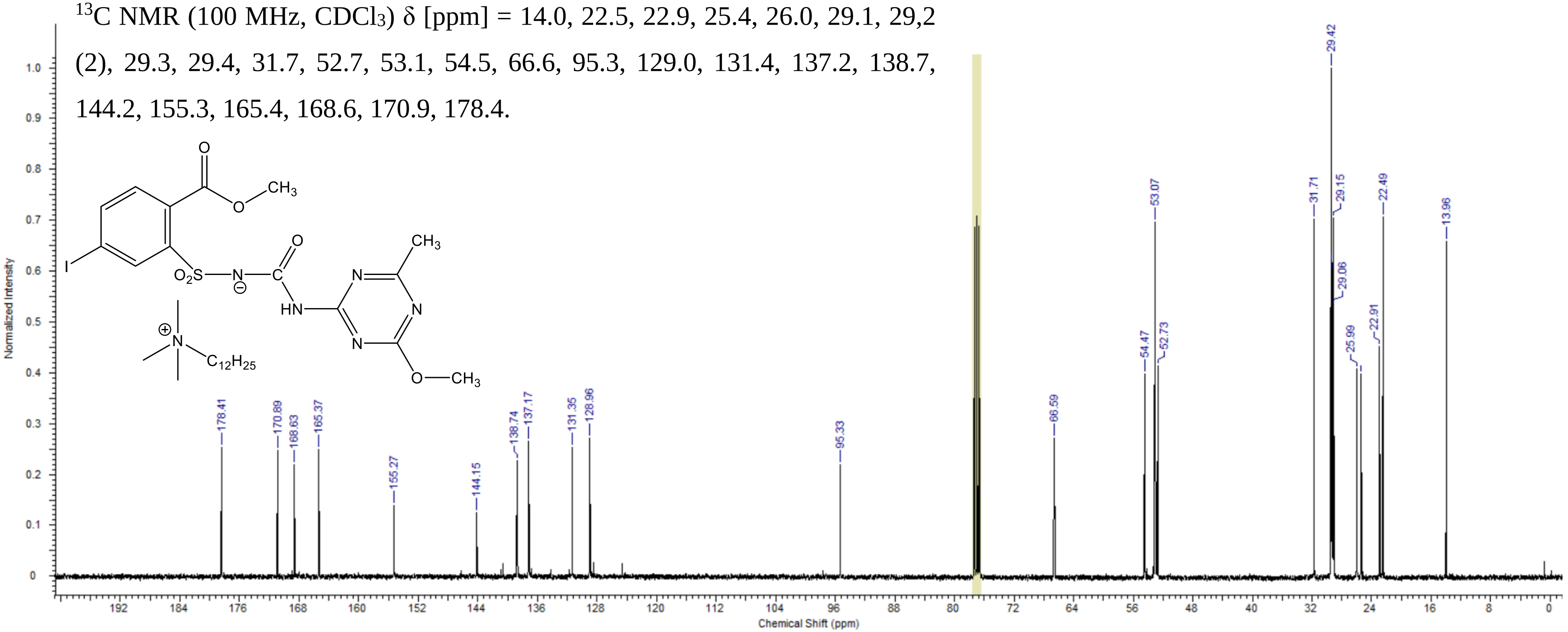

Figure S5. *UV spectrum of tetradecyltrimethylammonium ((5-iodo-2-(methoxycarbonyl)phenyl)sulfonyl)((4-methoxy-6-methyl-1,3,5-triazin-2-yl)carbamoyl)amidate (**2**).*

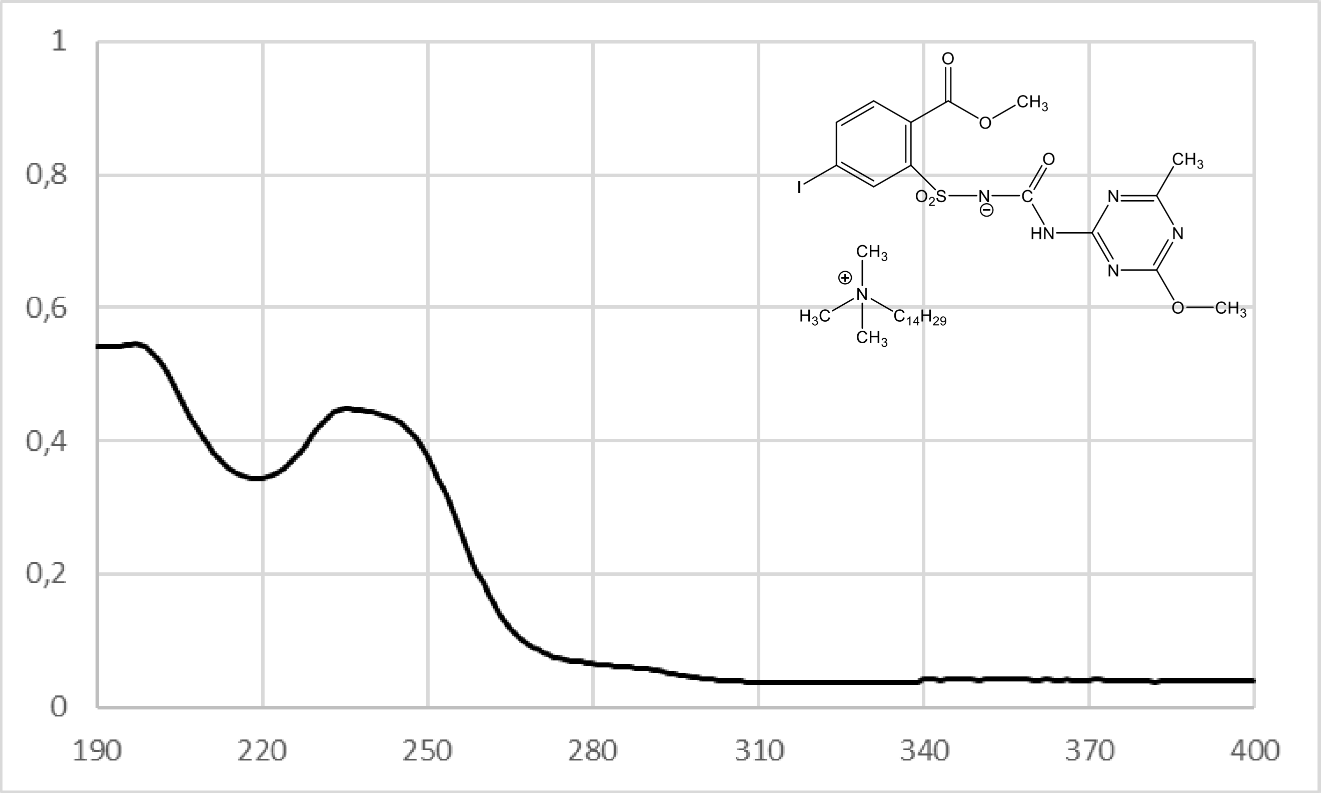

λ_{1max} *(water) = 197 nm;* λ_{2max} *(water) = 235 nm*

$\varepsilon_{\lambda 1max} = 3.07 \cdot 10^4\ dm^3\ mol^{-1}\ cm^{-1}$; $\varepsilon_{\lambda 2max} = 2.52 \cdot 10^4\ dm^3\ mol^{-1}\ cm^{-1}$

$\varepsilon_{\lambda 1max} / \varepsilon_{\lambda 2max} = 1.22$

Figure S6. *FT-IR spectrum of tetradecyltrimethylammonium ((5-iodo-2-(methoxycarbonyl)phenyl)sulfonyl)((4-methoxy-6-methyl-1,3,5-triazin-2-yl)carbamoyl)amidate (**2**).*

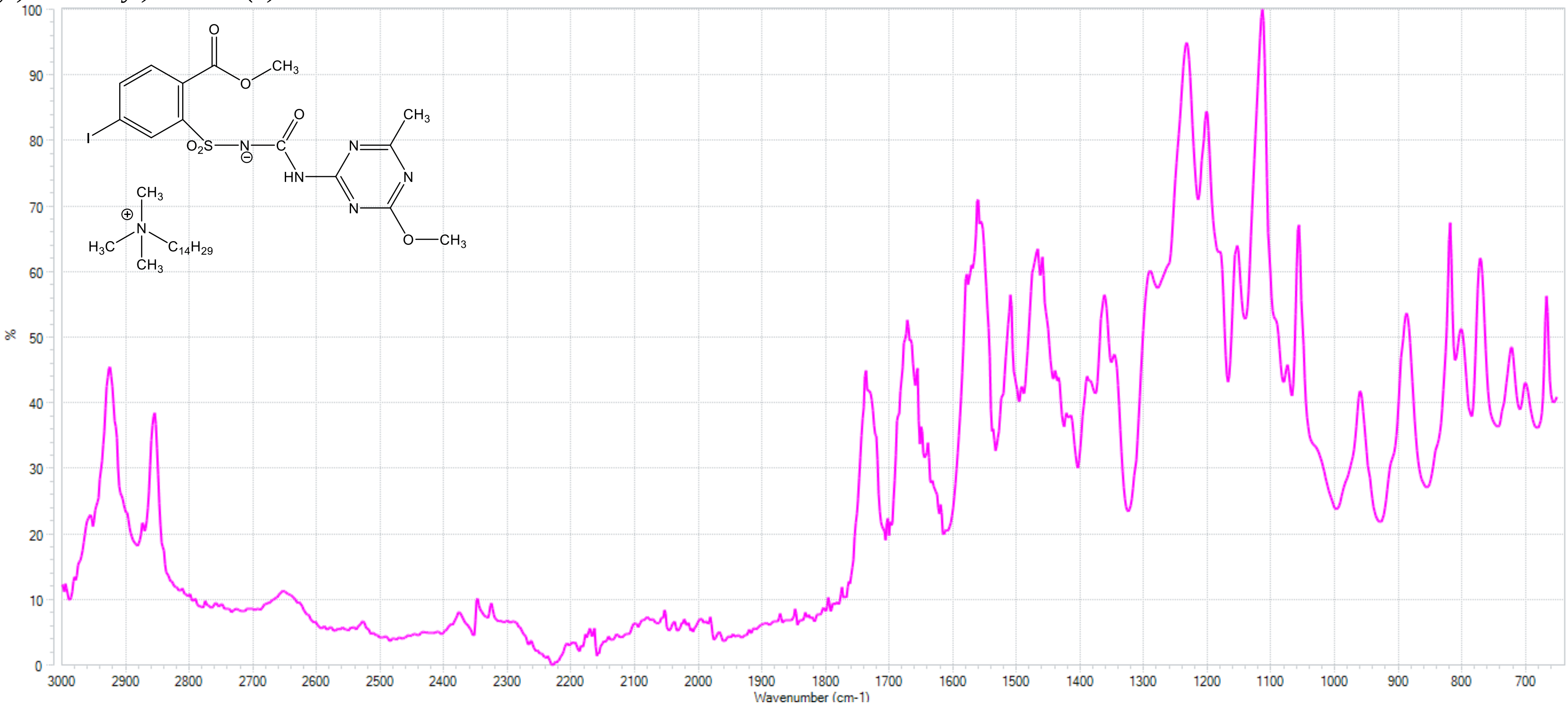

IR υ_{max} [cm^{-1}] = 2925, 2855, 1736, 1679, 1655, 1562, 1510, 1467, 1417, 1388, 1345, 1290, 1233, 1202, 1113, 1055, 887, 819, 773.

Figure S7. [1]*H NMR spectrum of tetradecyltrimethylammonium ((5-iodo-2-(methoxycarbonyl)phenyl)sulfonyl)((4-methoxy-6-methyl-1,3,5-triazin-2-yl)carbamoyl)amidate (**2**).*

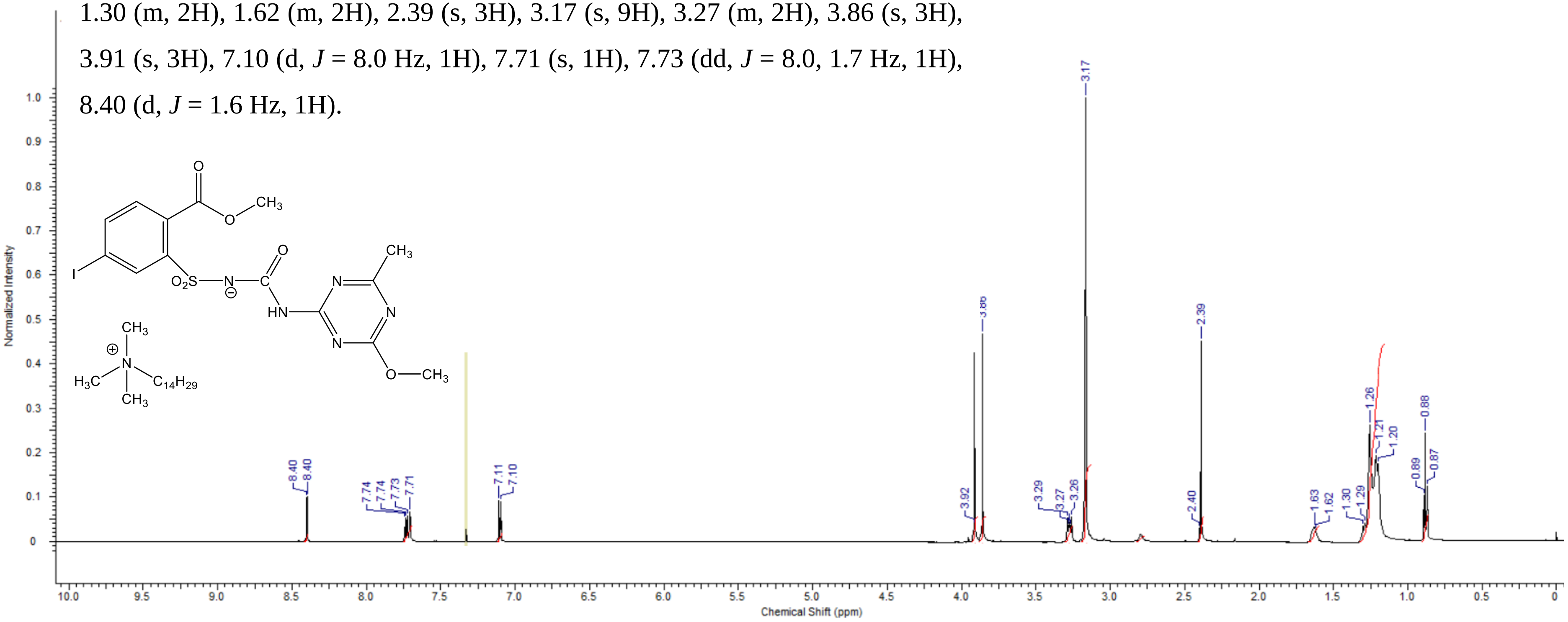

^{1}H NMR (400 MHz, $CDCl_3$) δ [ppm] = 0.88 (t, *J* = 7,0 Hz, 3H), 1.26 (m, 20H), 1.30 (m, 2H), 1.62 (m, 2H), 2.39 (s, 3H), 3.17 (s, 9H), 3.27 (m, 2H), 3.86 (s, 3H), 3.91 (s, 3H), 7.10 (d, *J* = 8.0 Hz, 1H), 7.71 (s, 1H), 7.73 (dd, *J* = 8.0, 1.7 Hz, 1H), 8.40 (d, *J* = 1.6 Hz, 1H).

Figure S8. *^{13}C NMR spectrum of tetradecyltrimethylammonium ((5-iodo-2-(methoxycarbonyl)phenyl)sulfonyl)((4-methoxy-6-methyl-1,3,5-triazin-2-yl)carbamoyl)amidate (**2**).*

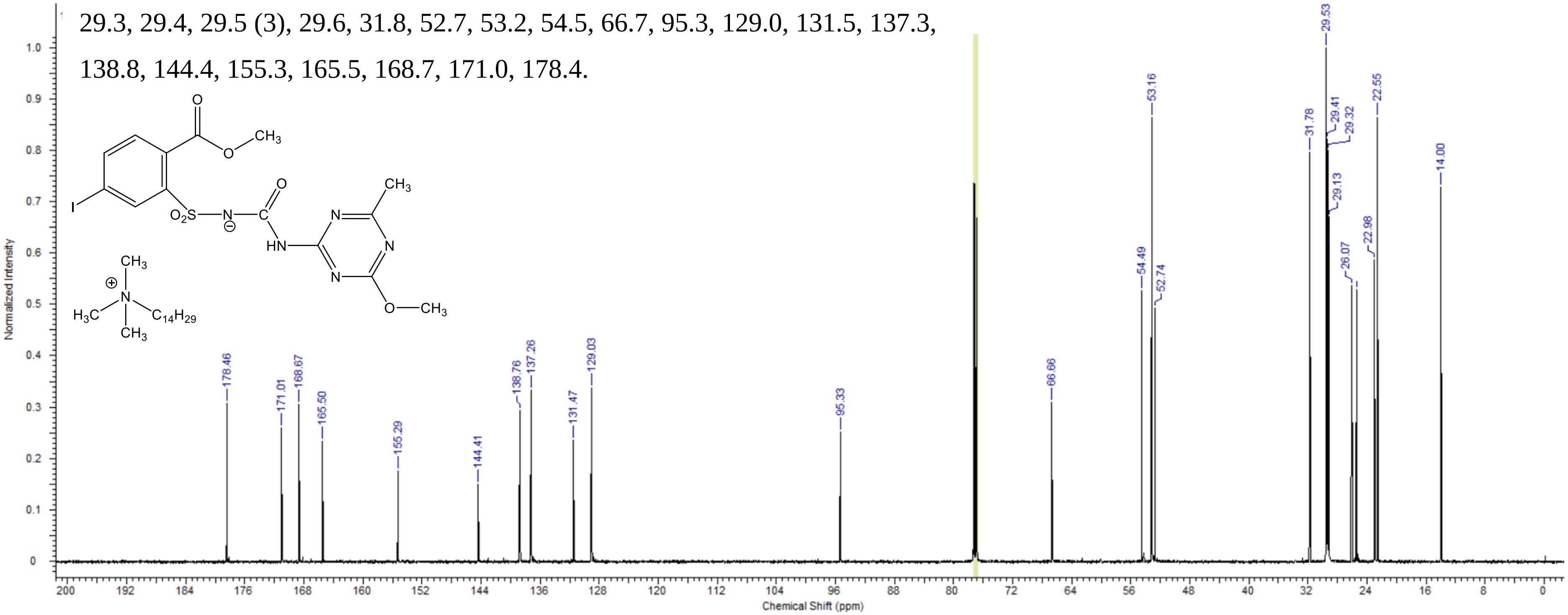

Figure S9. *UV spectrum of hexadecyltrimethylammonium ammonium ((5-iodo-2-(methoxycarbonyl)phenyl)sulfonyl)((4-methoxy-6-methyl-1,3,5-triazin-2-yl)carbamoyl)amidate (**3**).*

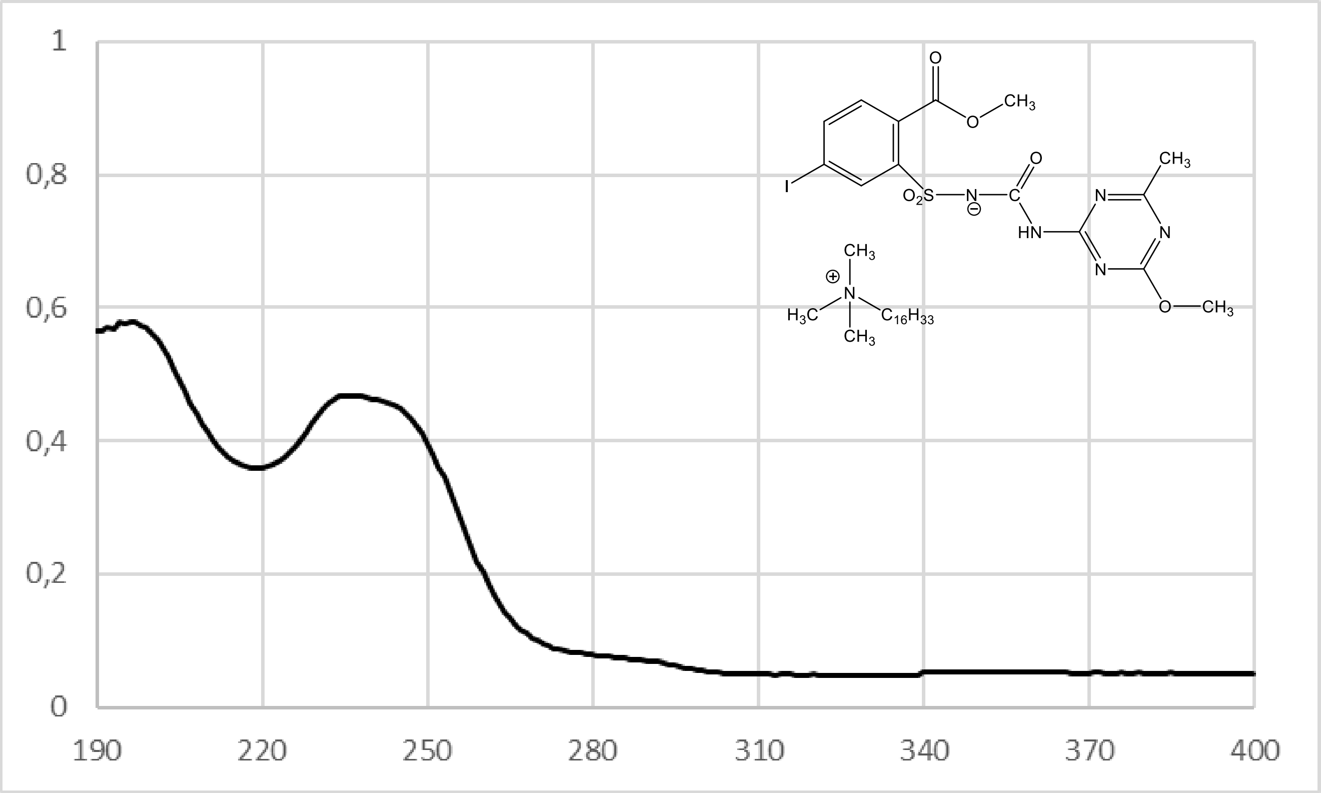

λ_{1max} *(water) = 197 nm;* λ_{2max} *(water) = 235 nm*

$\varepsilon_{\lambda 1max} = 3.19 \cdot 10^4\ dm^3\ mol^{-1}\ cm^{-1}$; $\varepsilon_{\lambda 2max} = 2.57 \cdot 10^4\ dm^3\ mol^{-1}\ cm^{-1}$

$\varepsilon_{\lambda 1max} / \varepsilon_{\lambda 2max} = 1.24$

Figure S10. *FT-IR spectrum of hexadecyltrimethylammonium ((5-iodo-2-(methoxycarbonyl)phenyl)sulfonyl)((4-methoxy-6-methyl-1,3,5-triazin-2-yl)carbamoyl)amidate (**3**).*

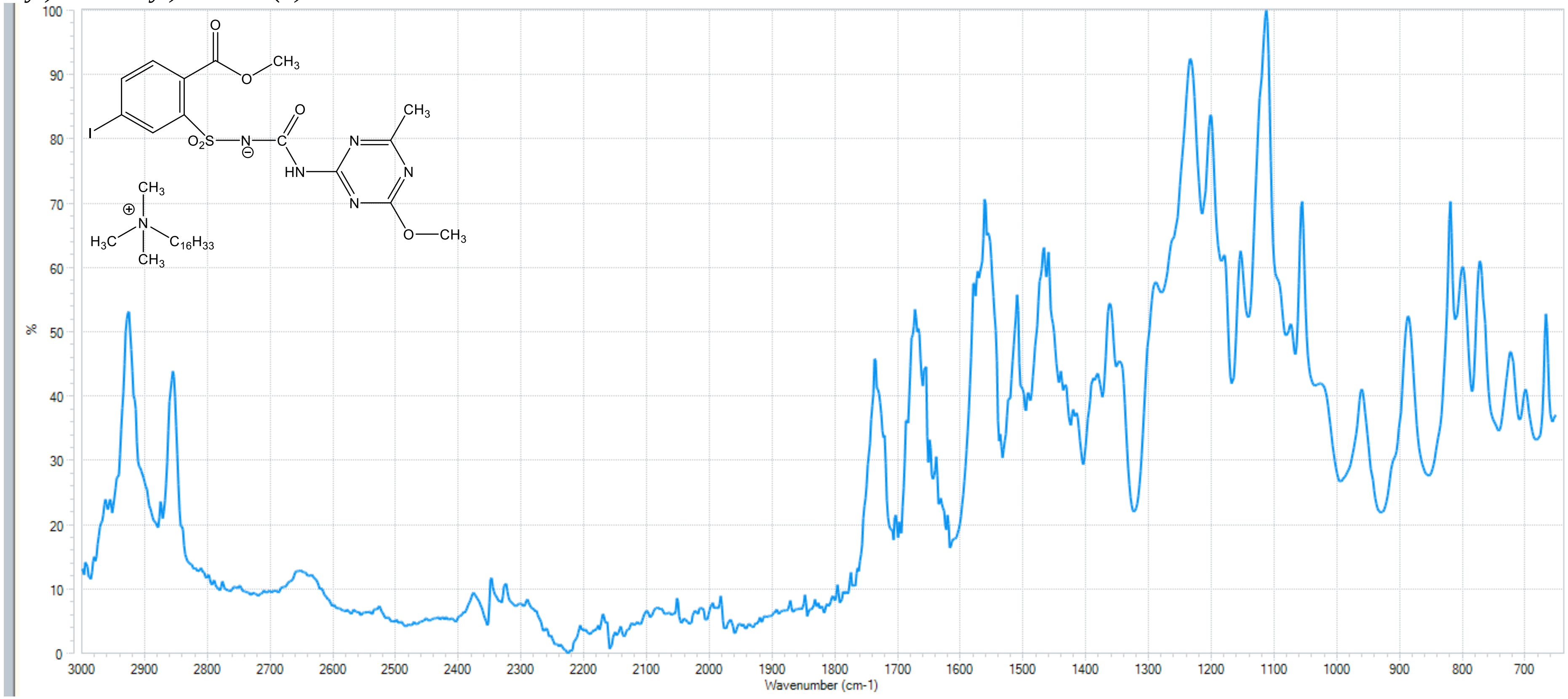

IR υ_{max} [cm^{-1}] = 2927, 2855, 1738, 1672, 1655, 1563, 1511, 1468, 1421, 1362, 1346, 1291, 1234, 1203, 1112, 1055, 887, 819, 772.

Figure S11. [1]*H NMR spectrum of hexadecyltrimethylammonium ((5-iodo-2-(methoxycarbonyl)phenyl)sulfonyl)((4-methoxy-6-methyl-1,3,5-triazin-2-yl)carbamoyl)amidate (**3**).*

^{1}H NMR (400 MHz, $CDCl_3$) δ [ppm] = 0.88 (t, *J* = 6,8 Hz, 3H), 1.26 (m, 26H), 1.63 (m, 2H), 2.39 (s, 3H), 3.18 (s, 9H), 3.28 (m, 2H), 3.86 (s, 3H), 3.91 (s, 3H), 7.11 (d, *J* = 7.9 Hz, 1H), 7.74 (m, 2H), 8.40 (d, *J* = 1.8 Hz, 1H).

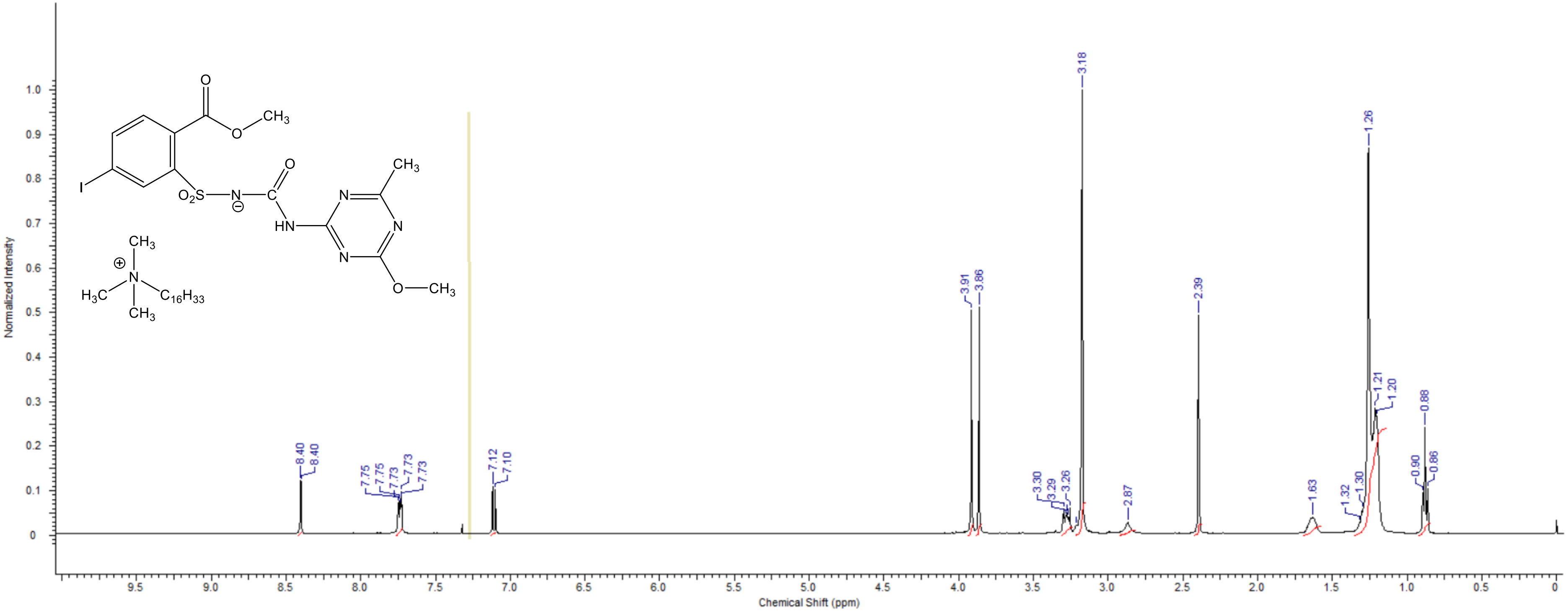

Figure S12. *^{13}C NMR spectrum of hexadecyltrimethylammonium ((5-iodo-2-(methoxycarbonyl)phenyl)sulfonyl)((4-methoxy-6-methyl-1,3,5-triazin-2-yl)carbamoyl)amidate (**3**).*

^{13}C NMR (100 MHz, $CDCl_3$) δ [ppm] = 14.0, 22.5, 22.9, 25.4, 26.0, 29.1, 29.2, 29.3, 29.4, 29.5 (2), 31.7, 52.7, 53.1, 54.5, 66.6, 95.3, 129.0, 131.3, 137.2, 138.7, 144.2, 155.3, 165.4, 168.6, 170.9, 178.4.

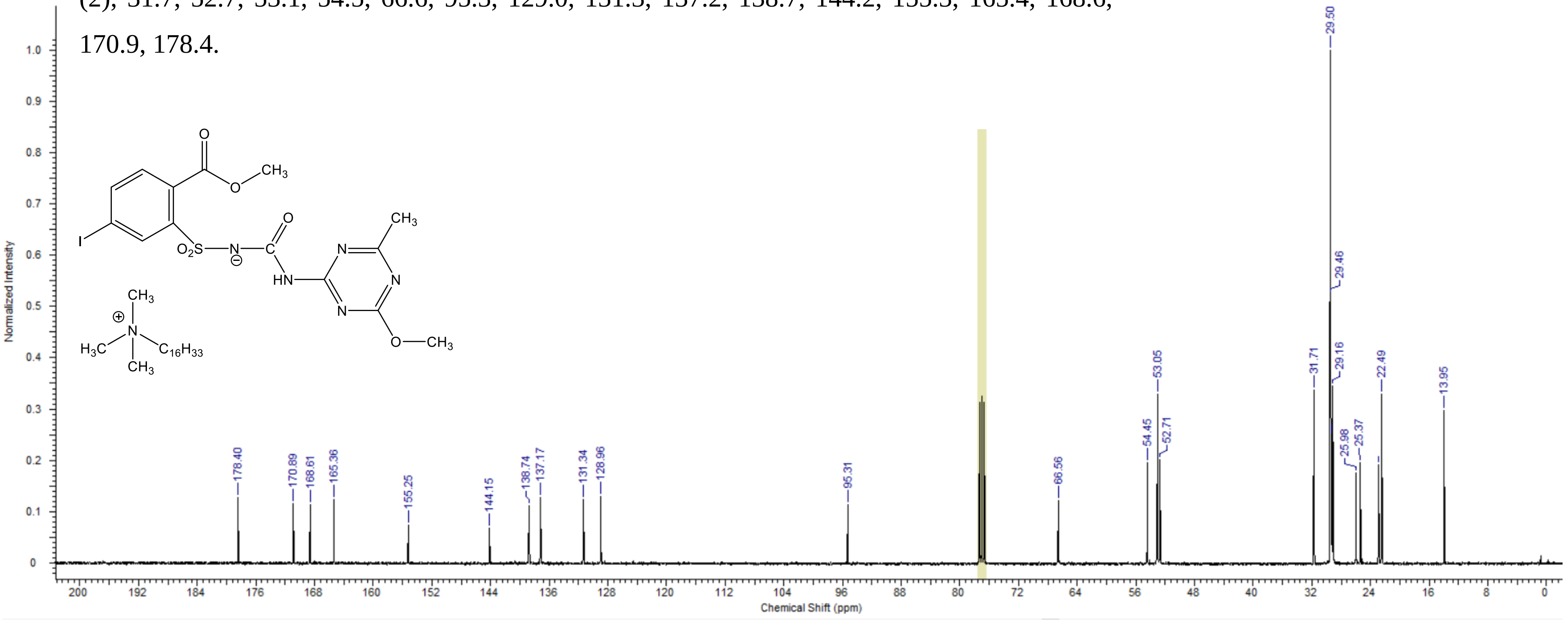

Figure S13. *UV spectrum of octadecyltrimethylammonium ((5-iodo-2-(methoxycarbonyl)phenyl)sulfonyl)((4-methoxy-6-methyl-1,3,5-triazin-2-yl)carbamoyl)amidate (**4**).*

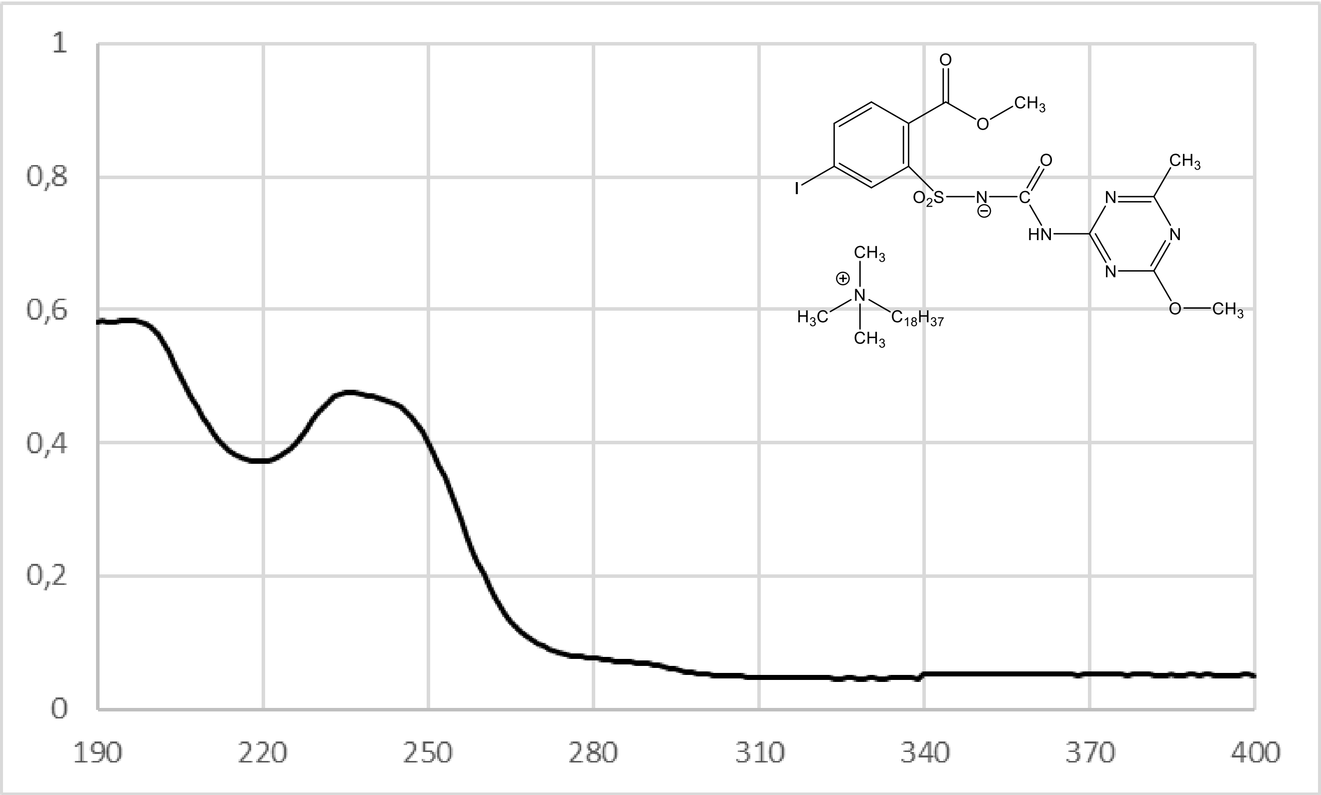

x

λ_{1max} *(water) = 197 nm;* λ_{2max} *(water) = 235 nm*

$\varepsilon_{\lambda 1max} = 2.89 \cdot 10^4\ dm^3\ mol^{-1}\ cm^{-1}$; $\varepsilon_{\lambda 2max} = 2.34 \cdot 10^4\ dm^3\ mol^{-1}\ cm^{-1}$

$\varepsilon_{\lambda 1max} / \varepsilon_{\lambda 2max} = 1.23$

***Figure S14**. FT-IR spectrum of octadecyltrimethylammonium ((5-iodo-2-(methoxycarbonyl)phenyl)sulfonyl)((4-methoxy-6-methyl-1,3,5-triazin-2-yl)carbamoyl)amidate (**4**).*

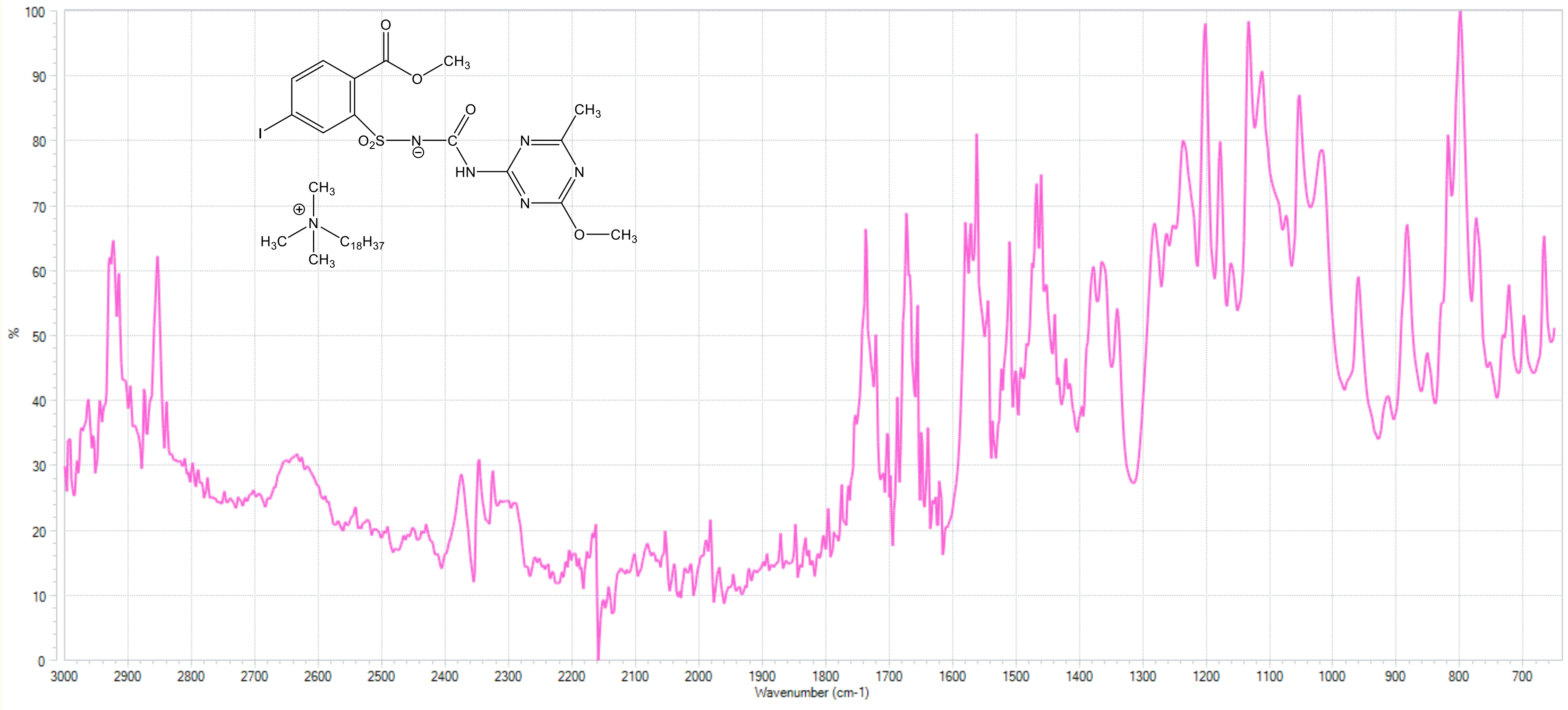

IR υmax [cm^{-1}] = 2923, 2853, 1738, 1671, 1655, 1562, 1510, 1467, 1421, 1365, 1340, 1281, 1236, 1200, 1111, 1054, 884, 819, 721.

Figure S15. [1]*H NMR spectrum of octadecyltrimethylammonium ((5-iodo-2-(methoxycarbonyl)phenyl)sulfonyl)((4-methoxy-6-methyl-1,3,5-triazin-2-yl)carbamoyl)amidate (**4**).*

^{1}H NMR (400 MHz, $CDCl_3$) δ [ppm] = 0.88 (t, *J* = 6,7 Hz, 3H), 1.25 (m, 30H), 1.64 (m, 2H), 2.39 (s, 3H), 3.18 (s, 9H), 3.28 (m, 2H), 3.86 (s, 3H), 3.91 (s, 3H), 7.11 (d, *J* = 7.9 Hz, 1H), 7.73 (m, 2H), 8.40 (m, 1H).

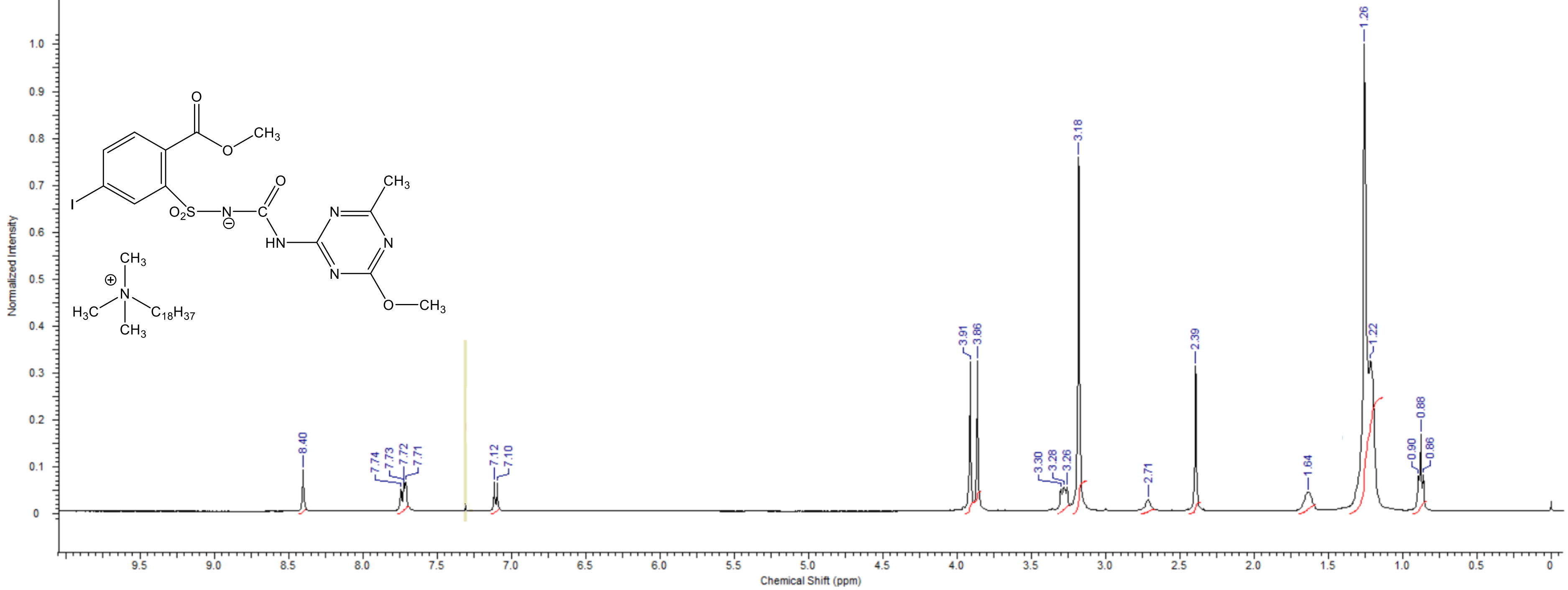

Figure S16. *^{13}C NMR spectrum of octadecyltrimethylammonium ((5-iodo-2-(methoxycarbonyl)phenyl)sulfonyl)((4-methoxy-6-methyl-1,3,5-triazin-2-yl)carbamoyl)amidate (**4**).*

^{13}C NMR (100 MHz, $CDCl_3$) δ [ppm] = 14.0, 22.5, 23.0, 25.4, 26.0, 29.1, 29.2, 29.3, 29.4, 29.5, 29.6, 31.8, 52.7, 53.1, 54.5, 66.6, 95.4, 129.0, 131.4, 137.2, 138.8, 144.2, 155.3, 165.4, 168.7, 170.9, 178.4.

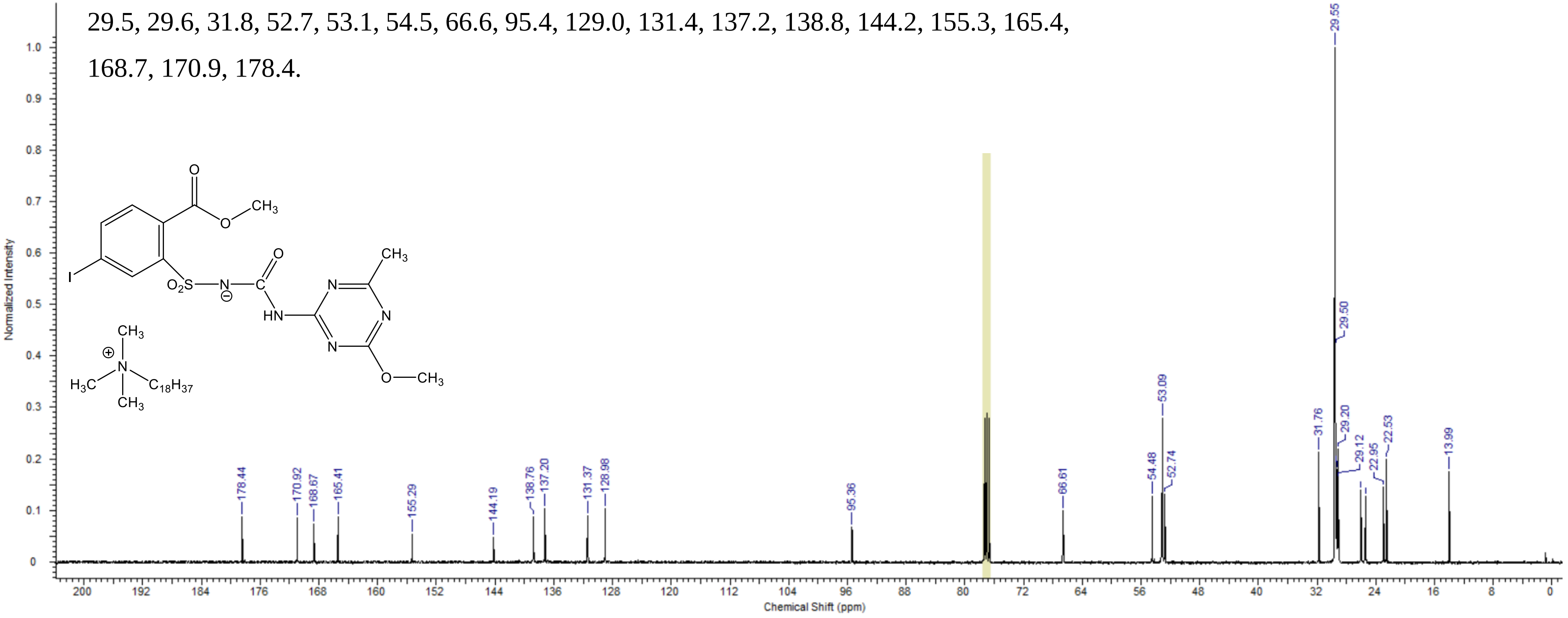

Figure S17. *UV spectrum of docosyltrimethylammonium ((5-iodo-2-(methoxycarbonyl)phenyl)sulfonyl)((4-methoxy-6-methyl-1,3,5-triazin-2-yl)carbamoyl)amidate (**5**).*

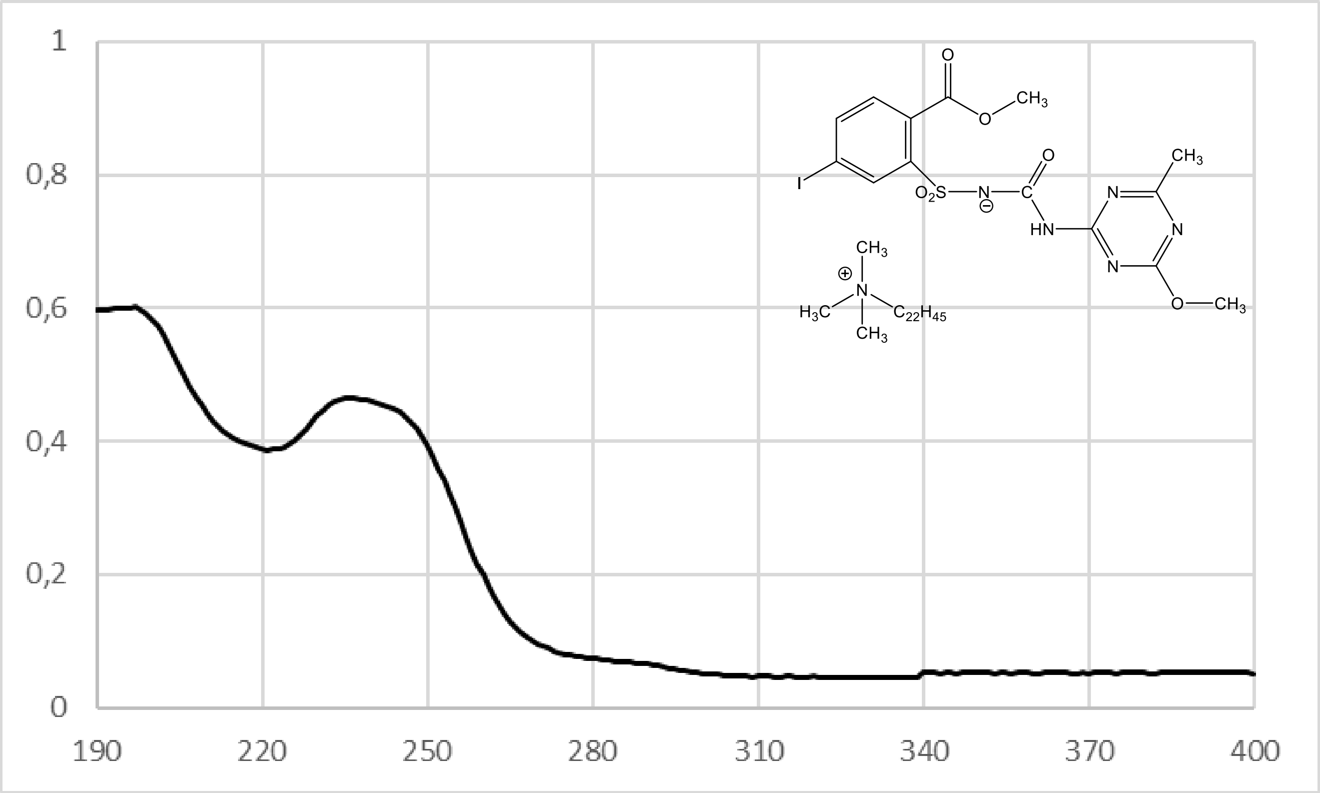

λ_{1max} *(water) = 197 nm;* λ_{2max} *(water) = 235 nm*

$\varepsilon_{\lambda 1max} = 3.13 \cdot 10^{4}\ dm^{3}\ mol^{-1}\ cm^{-1}$; $\varepsilon_{\lambda 2max} = 2.42 \cdot 10^{4}\ dm^{3}\ mol^{-1}\ cm^{-1}$

$\varepsilon_{\lambda 1max} / \varepsilon_{\lambda 2max} = 1.30$

Figure S18. *FT-IR spectrum of docosyltrimethylammonium ((5-iodo-2-(methoxycarbonyl)phenyl)sulfonyl)((4-methoxy-6-methyl-1,3,5-triazin-2-yl)carbamoyl)amidate (**5**).*

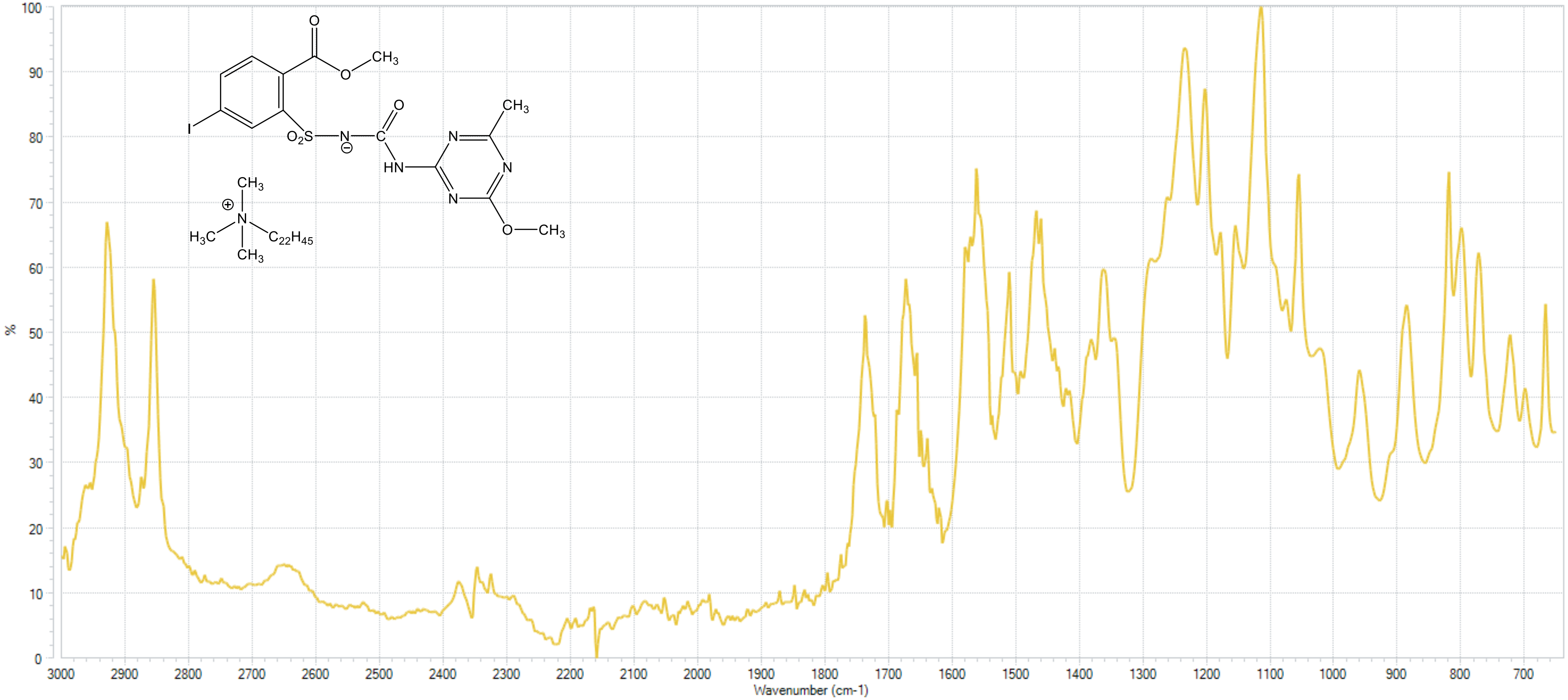

IR υ_{max} [cm^{-1}] = 2928, 2855, 1738, 1671, 1655, 1562, 1510, 1467, 1421, 1362, 1344, 1261, 1234, 1202, 1113, 1055, 885, 819, 722.

Figure S19. *^{1}H NMR spectrum of docosyltrimethylammonium ((5-iodo-2-(methoxycarbonyl)phenyl)sulfonyl)((4-methoxy-6-methyl-1,3,5-triazin-2-yl)carbamoyl)amidate (**5**).*

^{1}H NMR (300 MHz, $CDCl_3$) δ [ppm] = 0.88 (m, 3H), 1.25 (m, 38H), 1.64 (m, 2H), 2.39 (s, 3H), 3.18 (s, 9H), 3.27 (m, 2H), 3.86 (s, 3H), 3.91 (s, 3H), 7.10 (d, *J* = 8.0 Hz, 1H), 7.73 (m, 2H), 8.40 (d, *J* = 1.7 Hz, 1H).

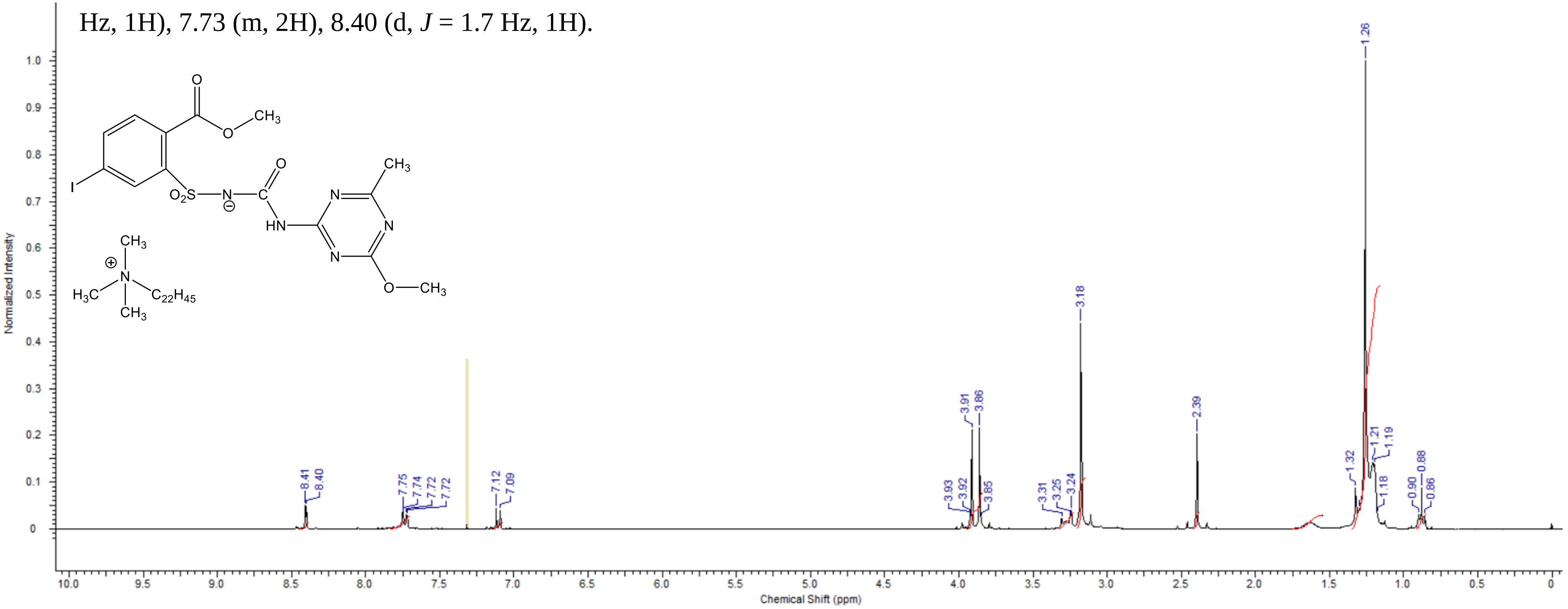

Figure S20. *^{13}C NMR spectrum of docosyltrimethylammonium ((5-iodo-2-(methoxycarbonyl)phenyl)sulfonyl)((4-methoxy-6-methyl-1,3,5-triazin-2-yl)carbamoyl)amidate (**5**).*

^{13}C NMR (75 MHz, $CDCl_3$) δ [ppm] = 14.0, 22.5, 22.9, 25.4, 26.0, 29.1, 29.2, 29.3, 29.4, 29.5, 31.7, 52.7, 53.1, 54.4, 66.6, 95.3, 129.0, 131.4, 137.2, 138.7, 144.3, 155.2, 165.4, 168.6, 171.0, 178.4.

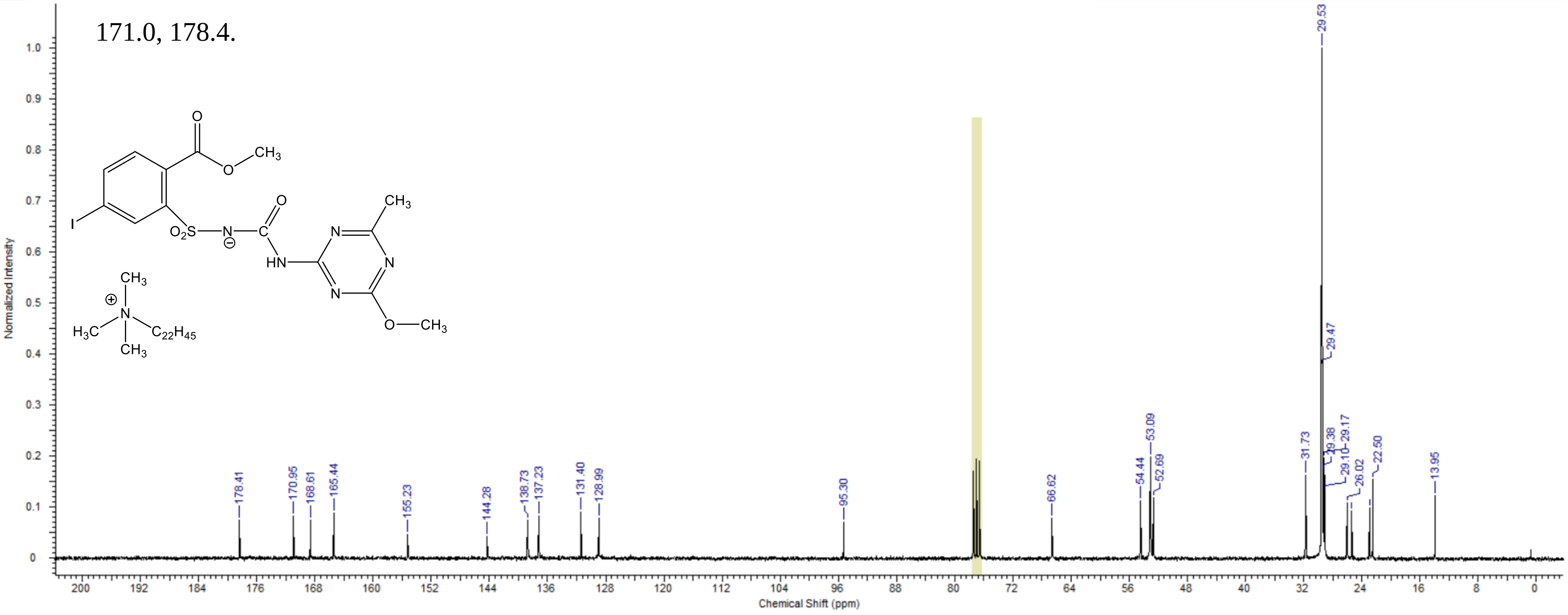

***Figure S21**. UV spectrum of dioctyldimethylammonium ((5-iodo-2-(methoxycarbonyl)phenyl)sulfonyl)((4-methoxy-6-methyl-1,3,5-triazin-2-yl)carbamoyl)amidate (**6**).*

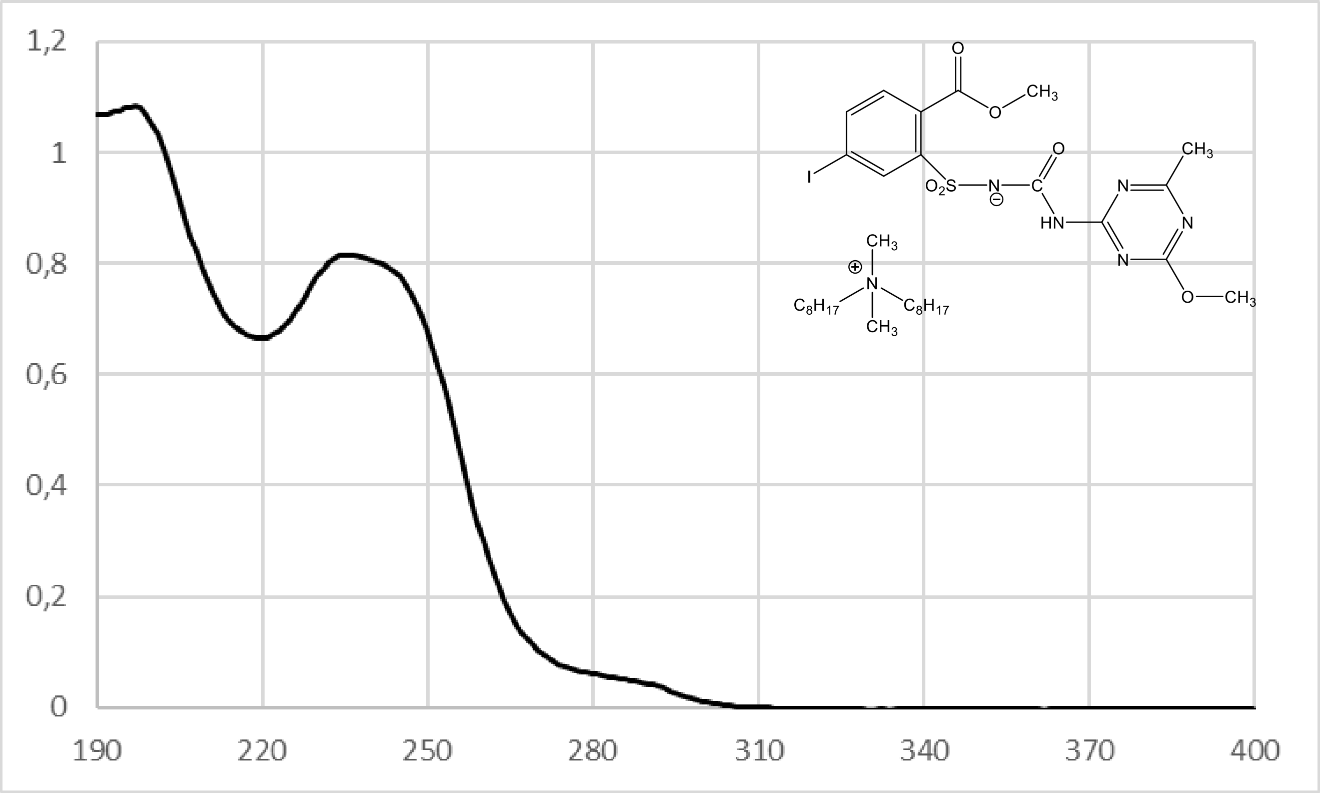

λ_{1max} *(water) = 197 nm;* λ_{2max} *(water) = 235 nm*

$\varepsilon_{\lambda 1max} = 2.98 \cdot 10^4$ $dm^3\ mol^{-1}\ cm^{-1}$; $\varepsilon_{\lambda 2max} = 2.24 \cdot 10^4$ $dm^3\ mol^{-1}\ cm^{-1}$

$\varepsilon_{\lambda 1max} / \varepsilon_{\lambda 2max} = 1.33$

***Figure S22**. FT-IR spectrum of dioctyldimethylammonium ((5-iodo-2-(methoxycarbonyl)phenyl)sulfonyl)((4-methoxy-6-methyl-1,3,5-triazin-2-yl)carbamoyl)amidate (**6**).*

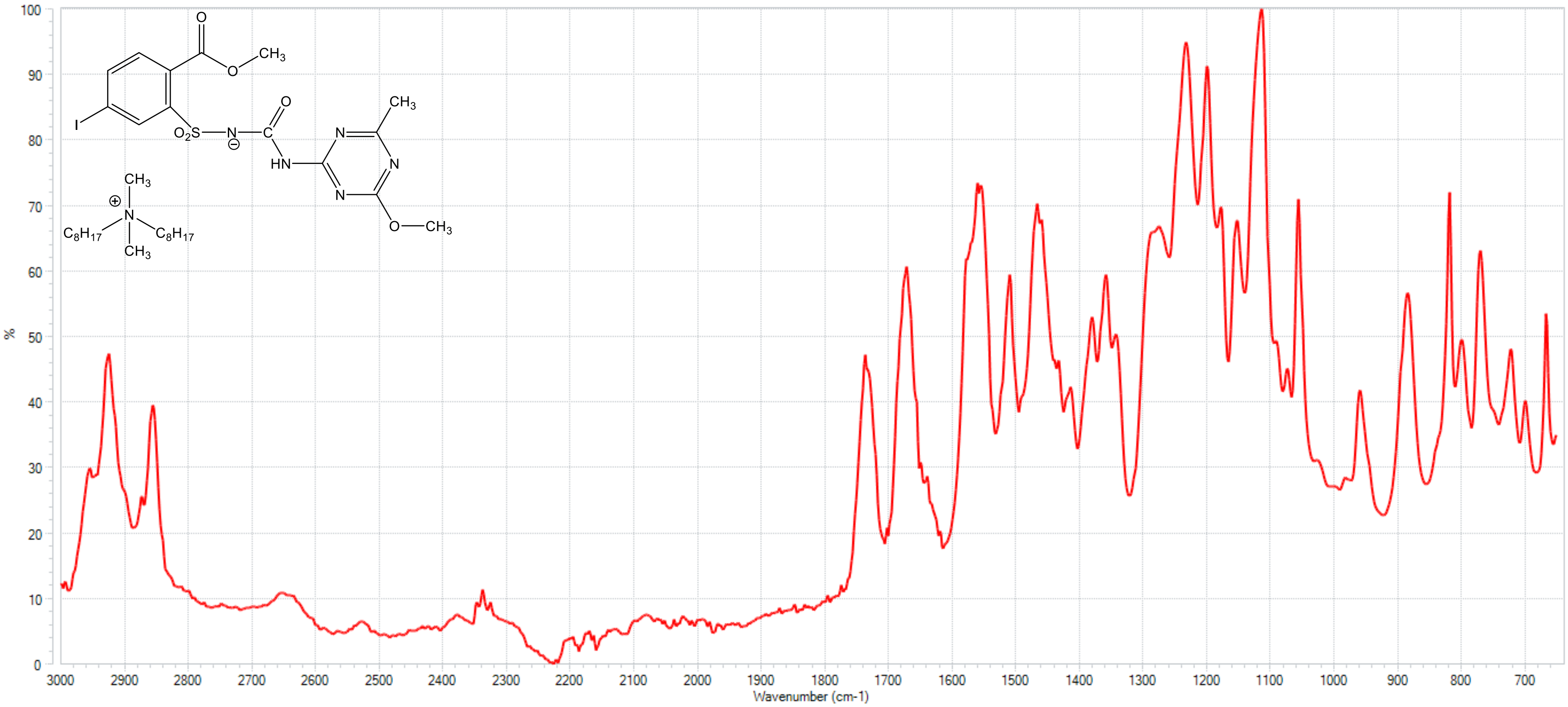

IR υmax [cm^{-1}] = 2924, 2854, 1735, 1672, 1578, 1560, 1509, 1468, 1431, 1359, 1343, 1274, 1233, 1200, 1113, 1056, 886, 819, 770.

Figure S23. *^{1}H NMR spectrum of dioctyldimethylammonium ((5-iodo-2-(methoxycarbonyl)phenyl)sulfonyl)((4-methoxy-6-methyl-1,3,5-triazin-2-yl)carbamoyl)amidate (**6**).*

^{1}H NMR (400 MHz, $CDCl_3$) δ [ppm] = 0.88 (t, *J* = 7.1 Hz, 6H), 1.25 (m, 20H), 1.61 (m, 4H), 2.40 (s, 3H), 3.13 (s, 6H), 3.25 (m, 4H), 3.87 (s, 3H), 3.92 (s, 3H), 7,10 (d, J = 8.0 Hz, 1H), 7.70 (s, 1H), 7.73 (dd, *J* = 8.0 Hz; 1.7 Hz, 1H), 8.42 (d, *J* = 1.7 Hz, 1H).

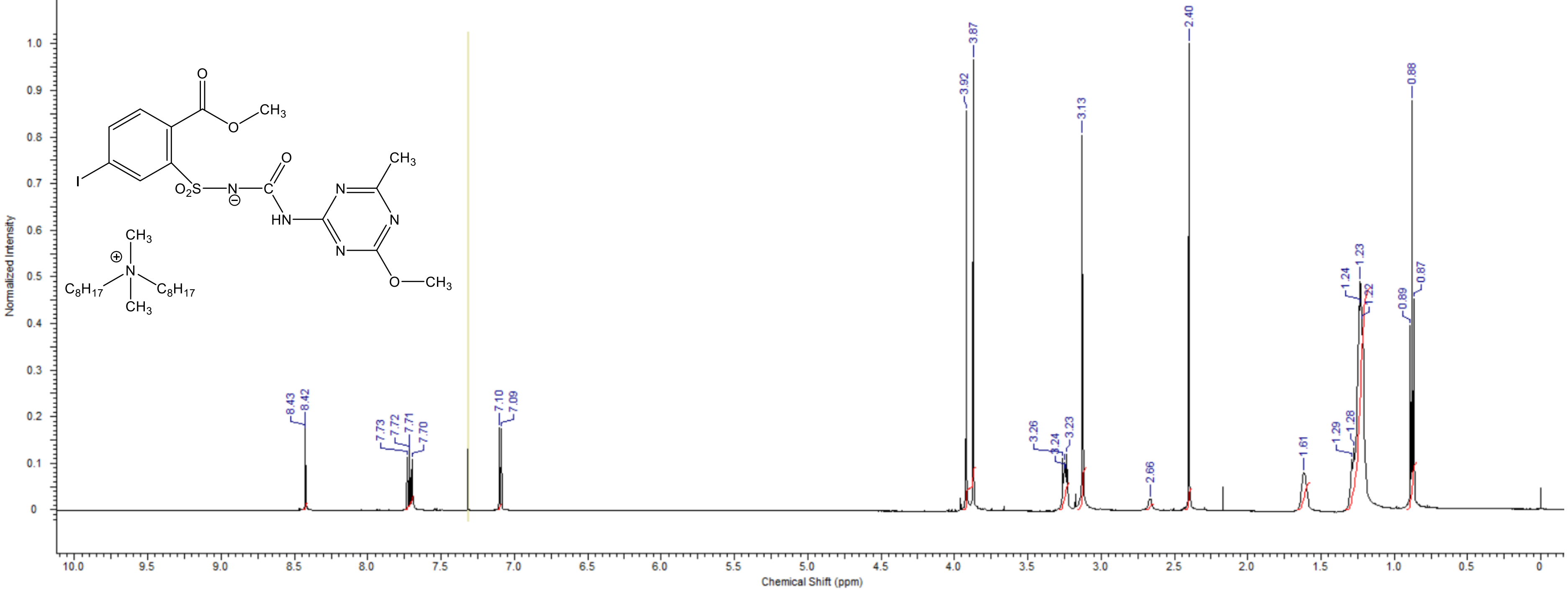

Figure S24. *^{13}C NMR spectrum of dioctyldimethylammonium ((5-iodo-2-(methoxycarbonyl)phenyl)sulfonyl)((4-methoxy-6-methyl-1,3,5-triazin-2-yl)carbamoyl)amidate (**6**).*

^{13}C NMR (100 MHz, $CDCl_3$) δ [ppm] = 13.9, 22.5 (2), 25.4, 26.1, 29.0, 29.1, 29.3 (2), 31.7, 50.9, 52.7, 54.4, 63.8, 95.3, 128.9, 131.5, 137.4, 138.6, 144.5, 155.1, 165.5, 168.7, 171.0, 178.4.

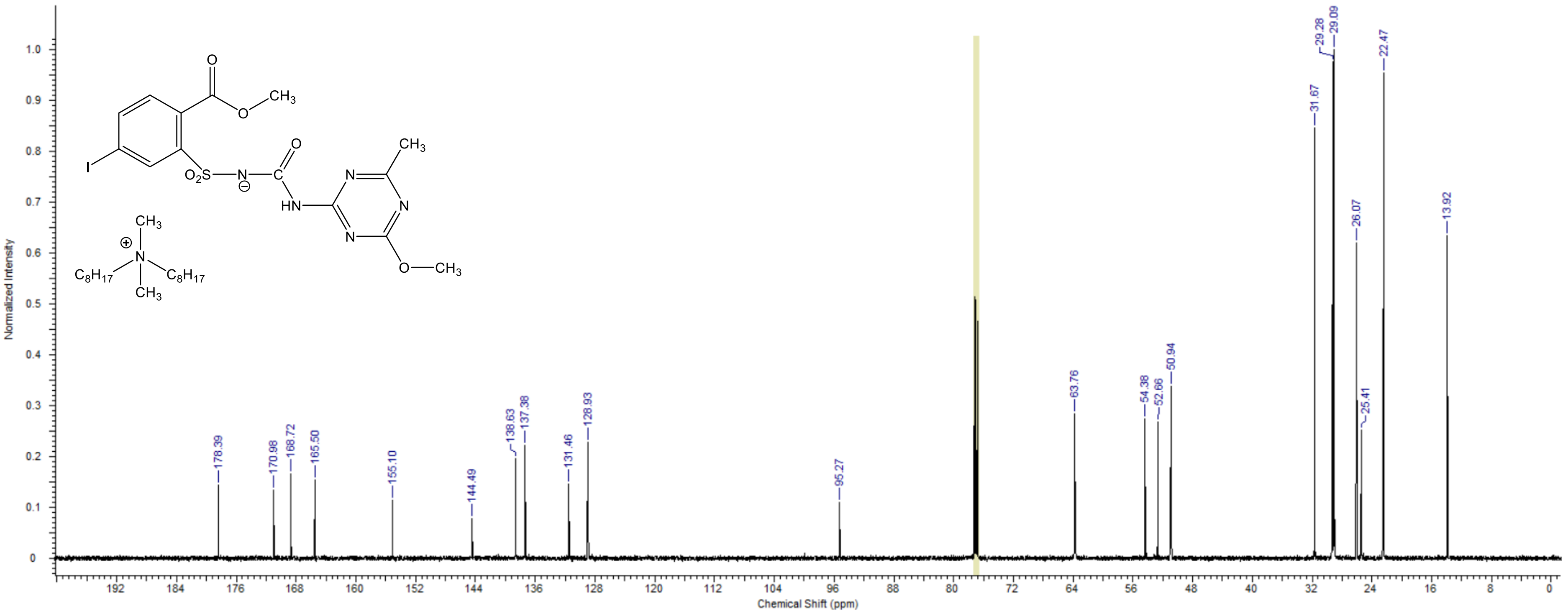

Figure S25. *UV spectrum of didecyldimethylammonium ((5-iodo-2-(methoxycarbonyl)phenyl)sulfonyl)((4-methoxy-6-methyl-1,3,5-triazin-2-yl)carbamoyl)amidate (**7**).*

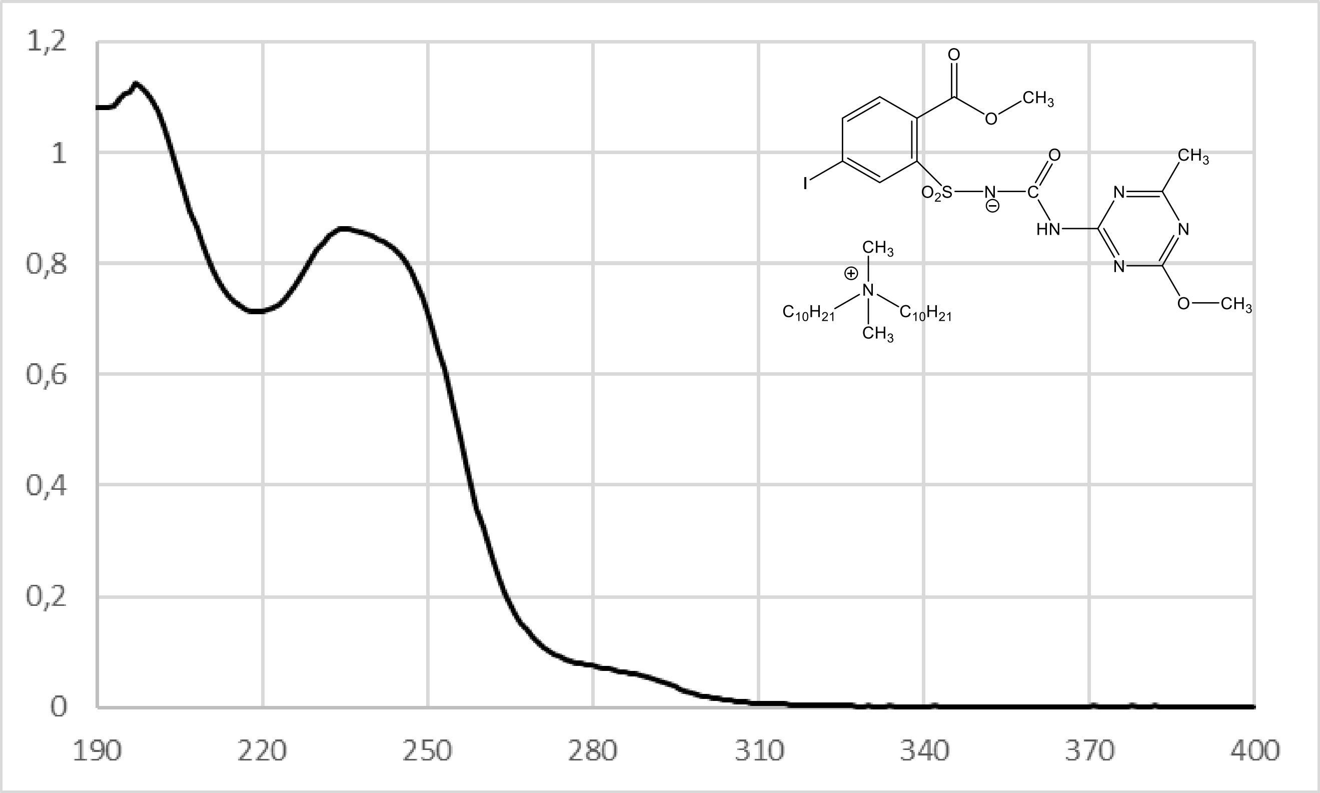

λ_{1max} (water) = 197 nm; λ_{2max} (water) = 235 nm

$\varepsilon_{\lambda 1max}$ = 3.33 10^4 dm^3 mol^{-1} cm^{-1}; $\varepsilon_{\lambda 2max}$ = 2.55 10^4 dm^3 mol^{-1} cm^{-1}

$\varepsilon_{\lambda 1max}$ /$\varepsilon_{\lambda 2max}$ = 1.30

Figure S26*. FT-IR spectrum of didecyldimethylammonium ((5-iodo-2-(methoxycarbonyl)phenyl)sulfonyl)((4-methoxy-6-methyl-1,3,5-triazin-2-yl)carbamoyl)amidate (****7****).*

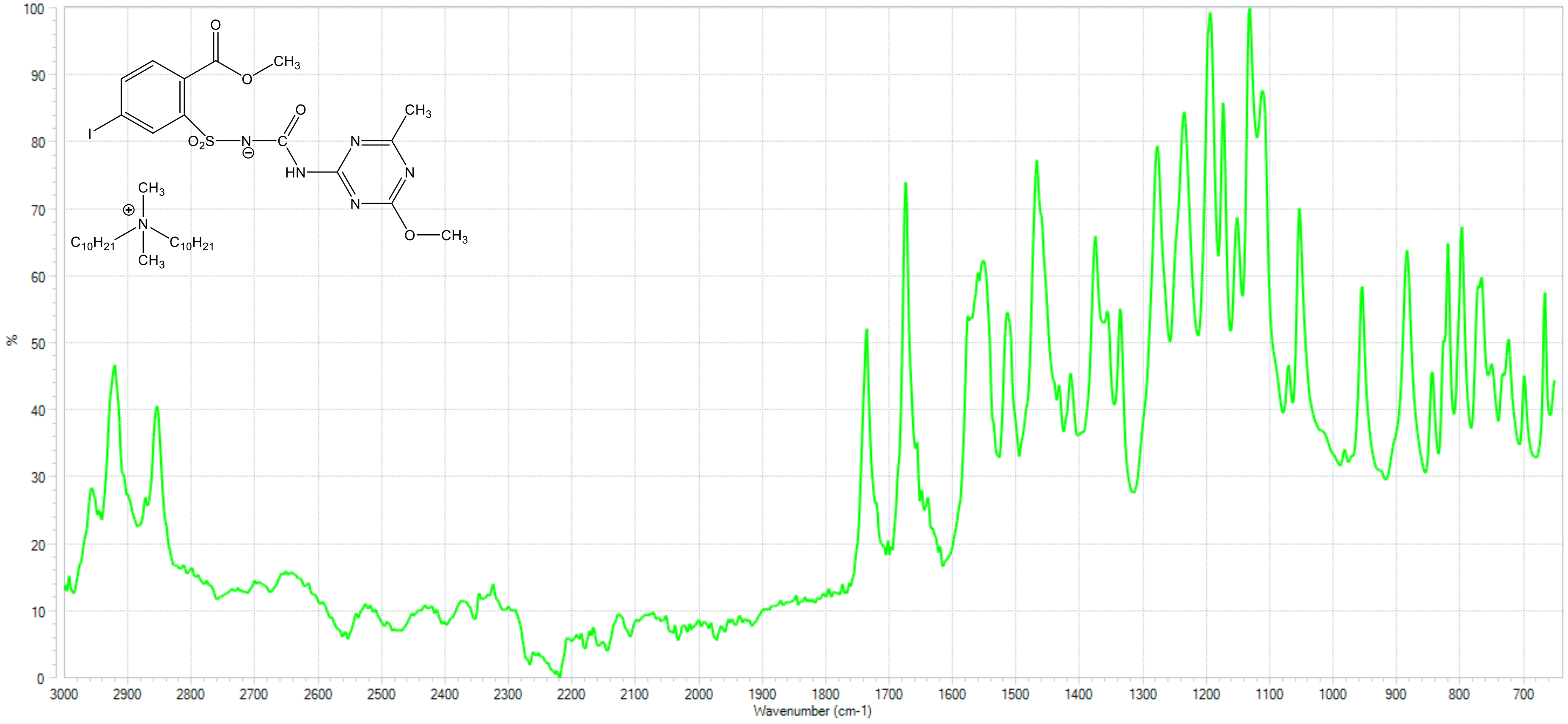

IR υ_{max} [cm^{-1}] = 2921, 2854, 1735, 1674, 1656, 1561, 1514, 1467, 1431, 1357, 1337, 1278, 1235, 1195, 1110, 1053, 883, 818, 772.

Figure S27. *^{1}H NMR spectrum of didecyldimethylammonium ((5-iodo-2-(methoxycarbonyl)phenyl)sulfonyl)((4-methoxy-6-methyl-1,3,5-triazin-2-yl)carbamoyl)amidate (**7**).*

^{1}H NMR (400 MHz, $CDCl_3$) δ [ppm] = 0.91 (m, 6H), 1.30 (m, 28H), 1.66 (m, 4H), 2.35 (s, 3H), 3.03 (s, 6H), 3.27 (m, 4H), 3.79 (s, 3H), 3.90 (s, 3H), 7,21 (d, J = 8.0 Hz, 1H), 7.73 (d, *J* = 7.9 Hz, 1H), 8.35 (d, *J* = 1.7 Hz, 1H).

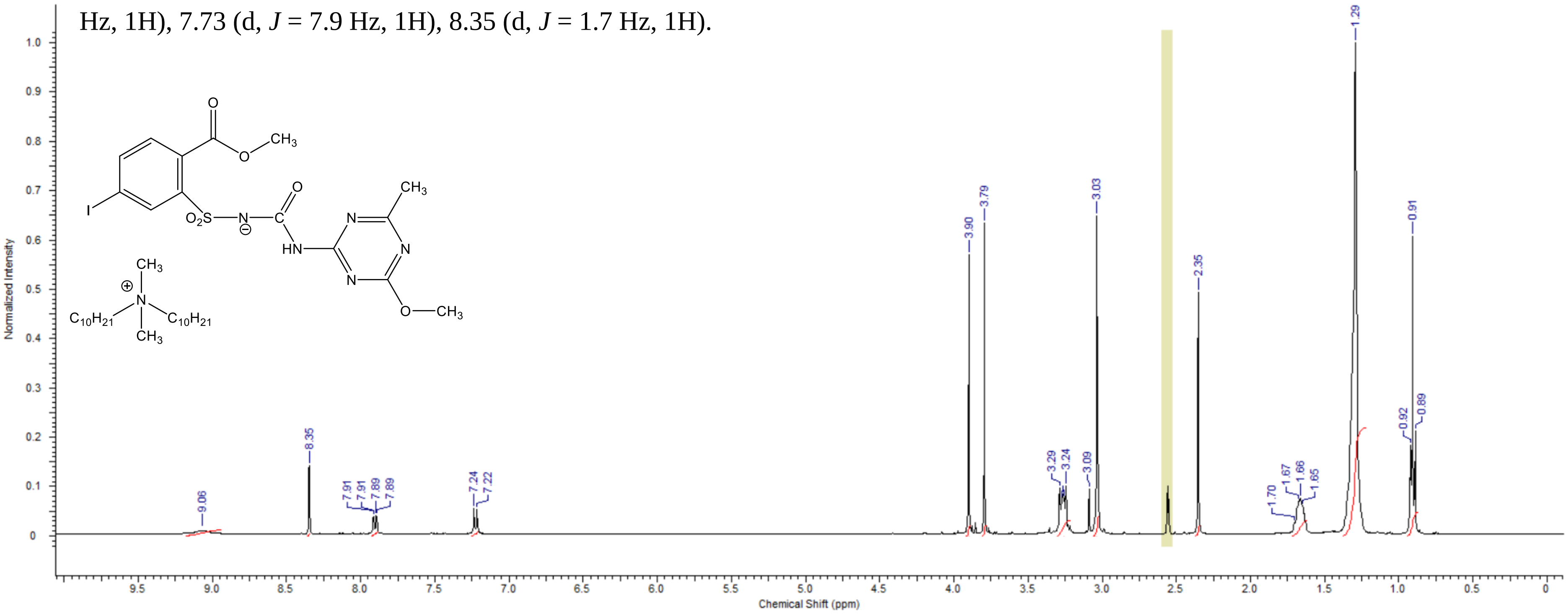

Figure S28. *^{13}C NMR spectrum of didecyldimethylammonium ((5-iodo-2-(methoxycarbonyl)phenyl)sulfonyl)((4-methoxy-6-methyl-1,3,5-triazin-2-yl)carbamoyl)amidate (**7**).*

^{13}C NMR (100 MHz, DMSO-d_6) δ [ppm] = 13.9, 21.7, 22.1, 25.1, 25.7, 28.5, 28.7, 28.7, 28.9, 31.3, 49.9, 52.4, 54.1, 62.8, 95.6, 129.0, 131.2, 137.3, 138.3, 144.8, 154.6, 165.5, 165.6, 168.1, 170.6, 177.4.

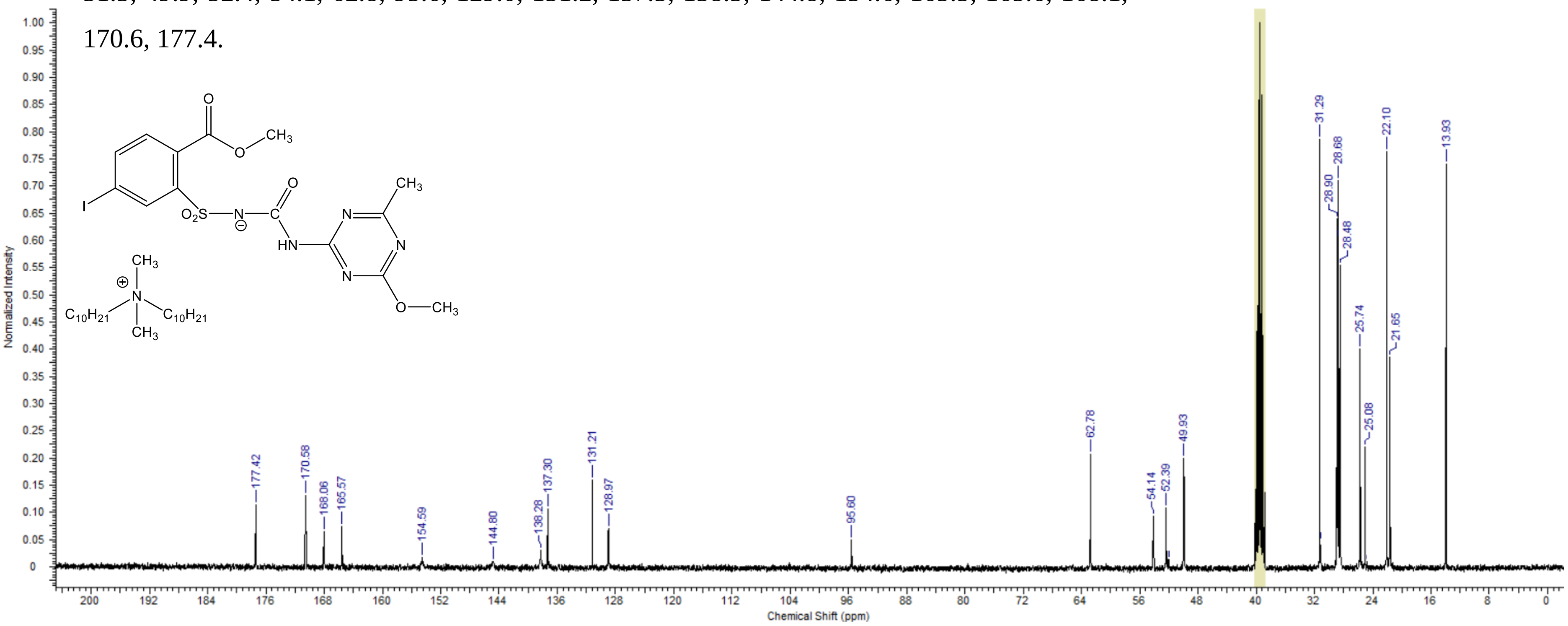

***Figure S29**. UV spectrum of di(hydrogenated tallow)dimethylammonium ((5-iodo-2-(methoxycarbonyl)phenyl)sulfonyl)((4-methoxy-6-methyl-1,3,5-triazin-2-yl)carbamoyl)amidate (**8**).*

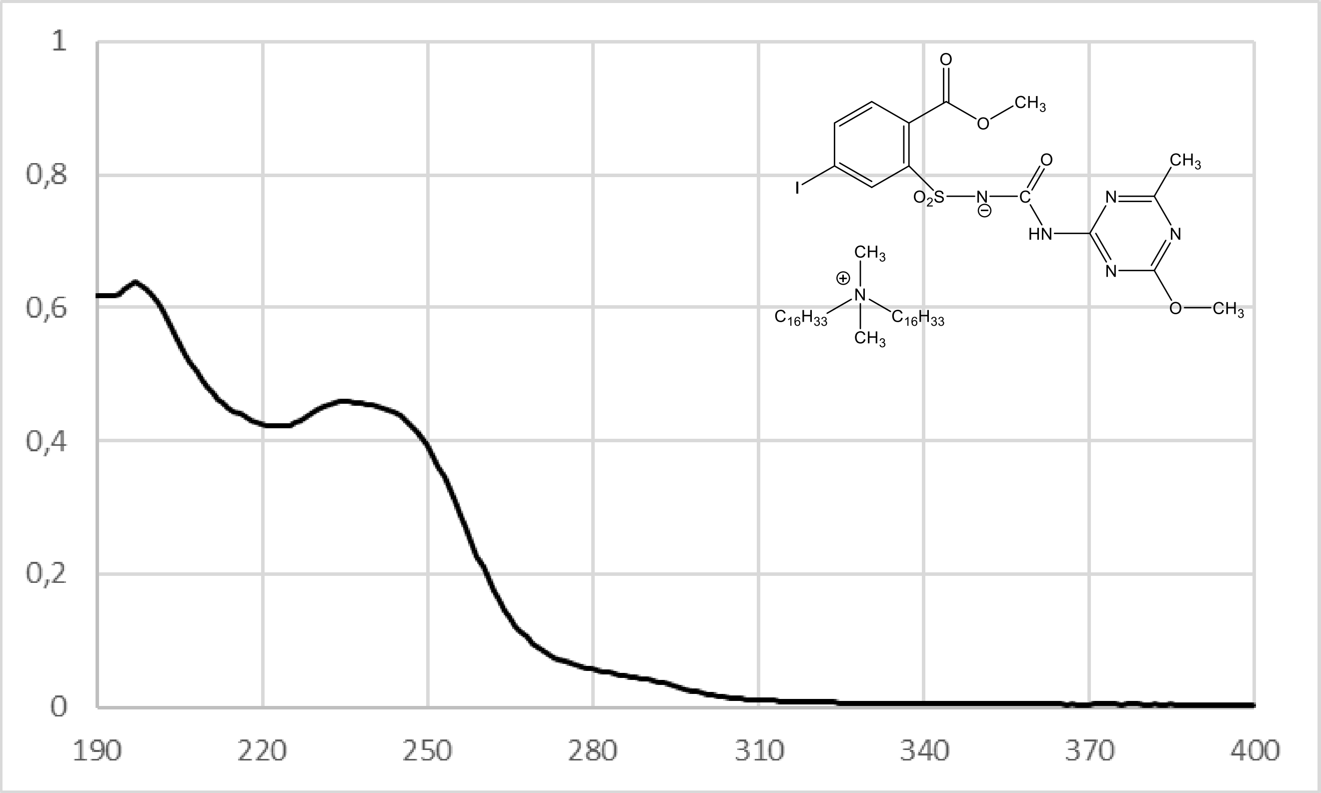

λ_{1max} (water) = 197 nm; λ_{2max} (water) = 235 nm

$\varepsilon_{\lambda 1max}$ = 3.21 $\cdot 10^4$ dm^3 mol^{-1} cm^{-1}; $\varepsilon_{\lambda 2max}$ = 2.31 $\cdot 10^4$ dm^3 mol^{-1} cm^{-1}

$\varepsilon_{\lambda 1max}$ /$\varepsilon_{\lambda 2max}$ = 1.39

Figure S30. *FT-IR spectrum of di(hydrogenated tallow)dimethylammonium ((5-iodo-2-(methoxycarbonyl)phenyl)sulfonyl)((4-methoxy-6-methyl-1,3,5-triazin-2-yl)carbamoyl)amidate (**8**).*

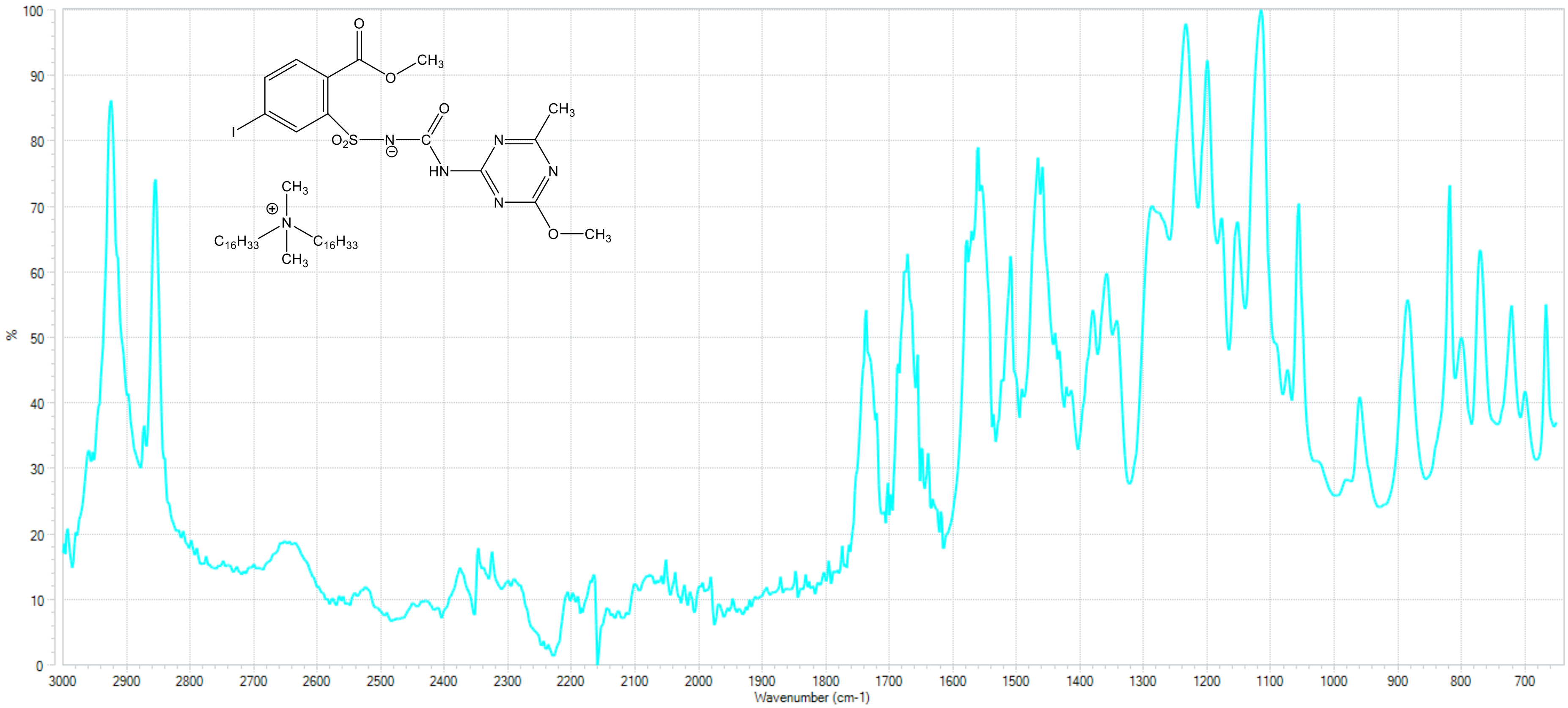

IR υ_{max} [cm^{-1}] = 2924, 2856, 1735, 1672, 1656, 1561, 1510, 1467, 1431, 1359, 1343, 1289, 1235, 1200, 1116, 1054, 885, 818, 770.

Figure S31. *^{1}H NMR spectrum of di(hydrogenated tallow)dimethylammonium ((5-iodo-2-(methoxycarbonyl)phenyl)sulfonyl)((4-methoxy-6-methyl-1,3,5-triazin-2-yl)carbamoyl)amidate (**8**).*

^{1}H NMR (300 MHz, $CDCl_3$) δ [ppm] = 0.88 (m, 6H), 1.26 (m, 52H), 1.61 (m, 4H), 2.40 (s, 3H), 3.13 (s, 6H), 3.24 (m, 4H), 3.87 (s, 3H), 3.91 (s, 3H), 7,10 (d, J = 8.0 Hz, 1H), 7.70 (d, J = 1.7 Hz, 1H), 7.73 (d, J = 1.7 Hz, 1H), 8.42 (d, J = 1.7 Hz, 1H).

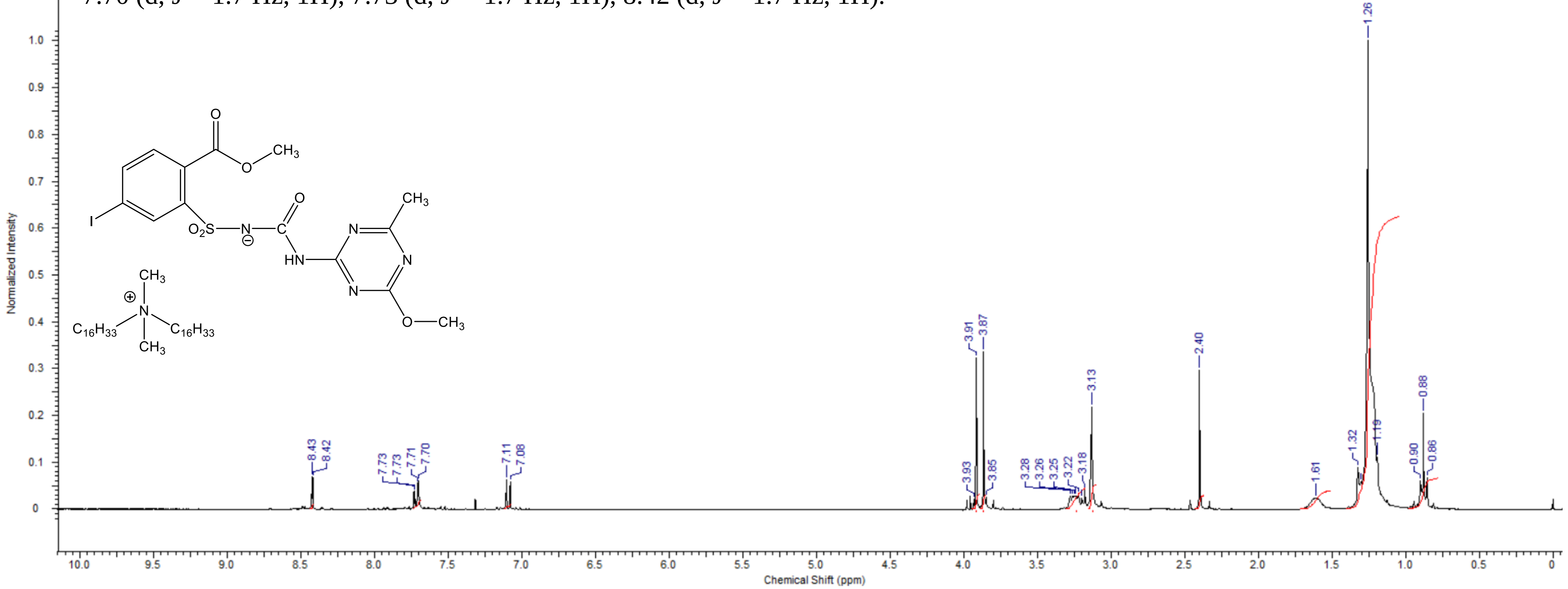

Figure S32. *^{13}C NMR spectrum of di(hydrogenated tallow)dimethylammonium ((5-iodo-2-(methoxycarbonyl)phenyl)sulfonyl)((4-methoxy-6-methyl-1,3,5-triazin-2-yl)carbamoyl)amidate (**8**).*

^{13}C NMR (75 MHz, $CDCl_3$) δ [ppm] = 13.9, 22.5, 25.4, 26.1, 29.0, 29.1, 29.2, 29.3, 29.4, 29.5 (2), 31.8, 50.9, 52.6, 54.4, 63.7, 95.3, 128.9, 131.5, 137.4, 138.6, 144.5, 155.1, 165.5, 168.7, 171.0, 178.4.

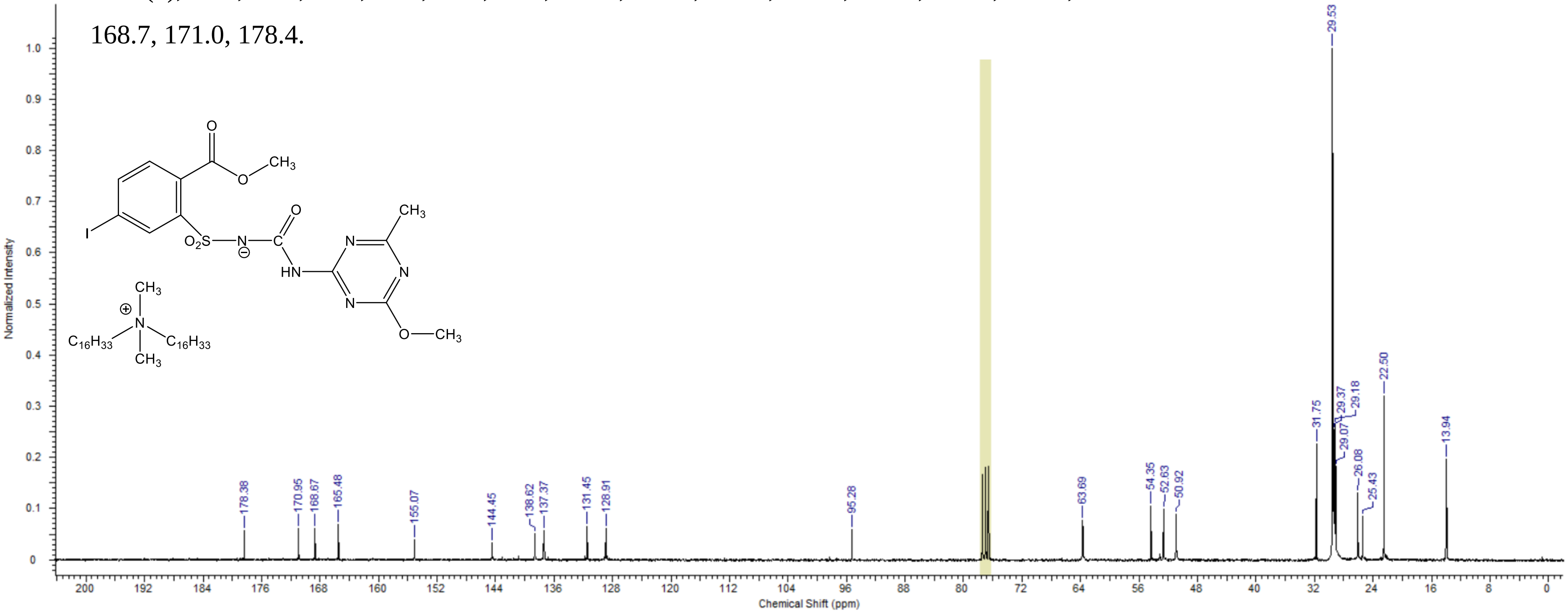

Figure S33. *The FT-IR spectra of product with mirystyltrimethylammonium cation (**2**) with numbering of the most characteristic vibrations*

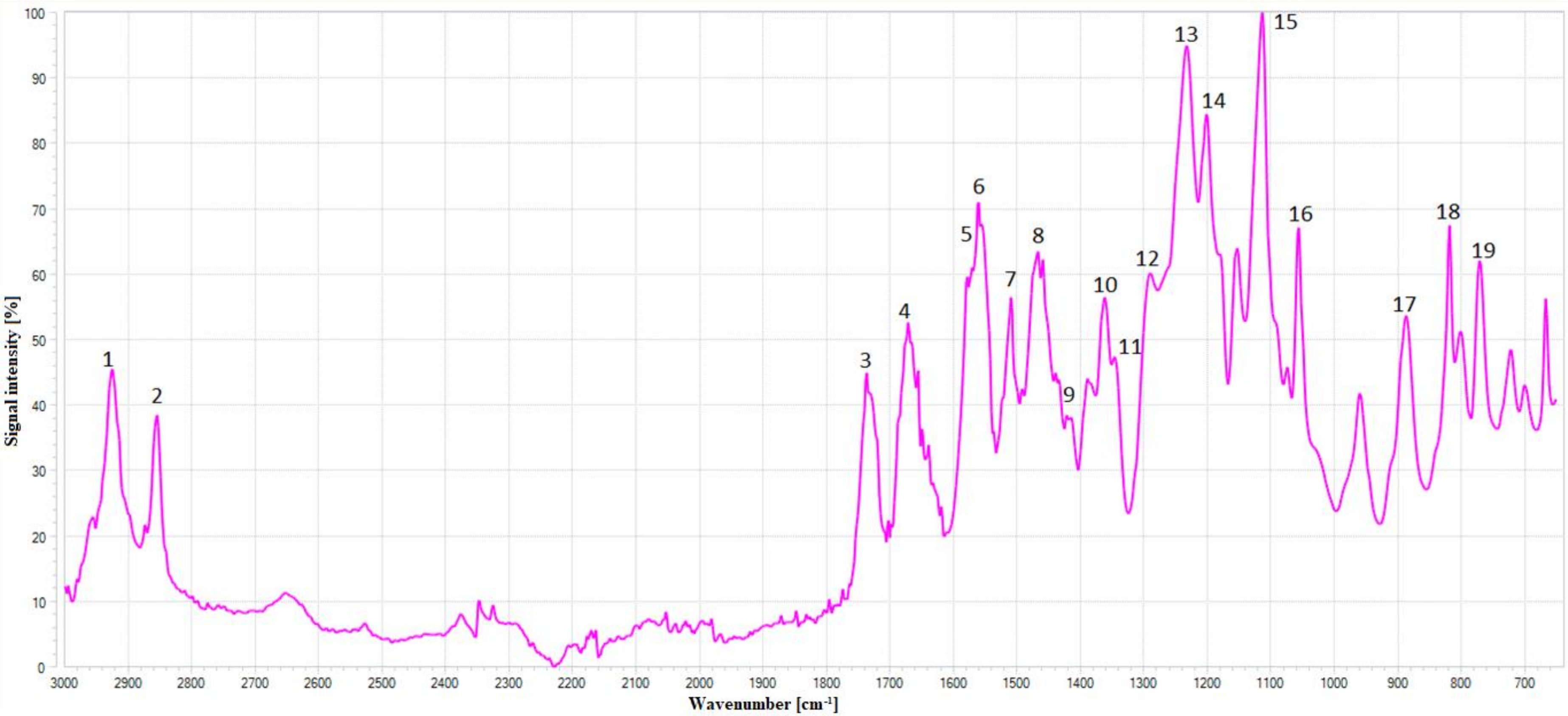

Figure S34*. The comparison between FT-IR spectra of ILs comprising alkyltrimethylammonium cation (****1-5****) compared to iodosulfuron-methyl sodium salt* ***[Na][ISM]****.*

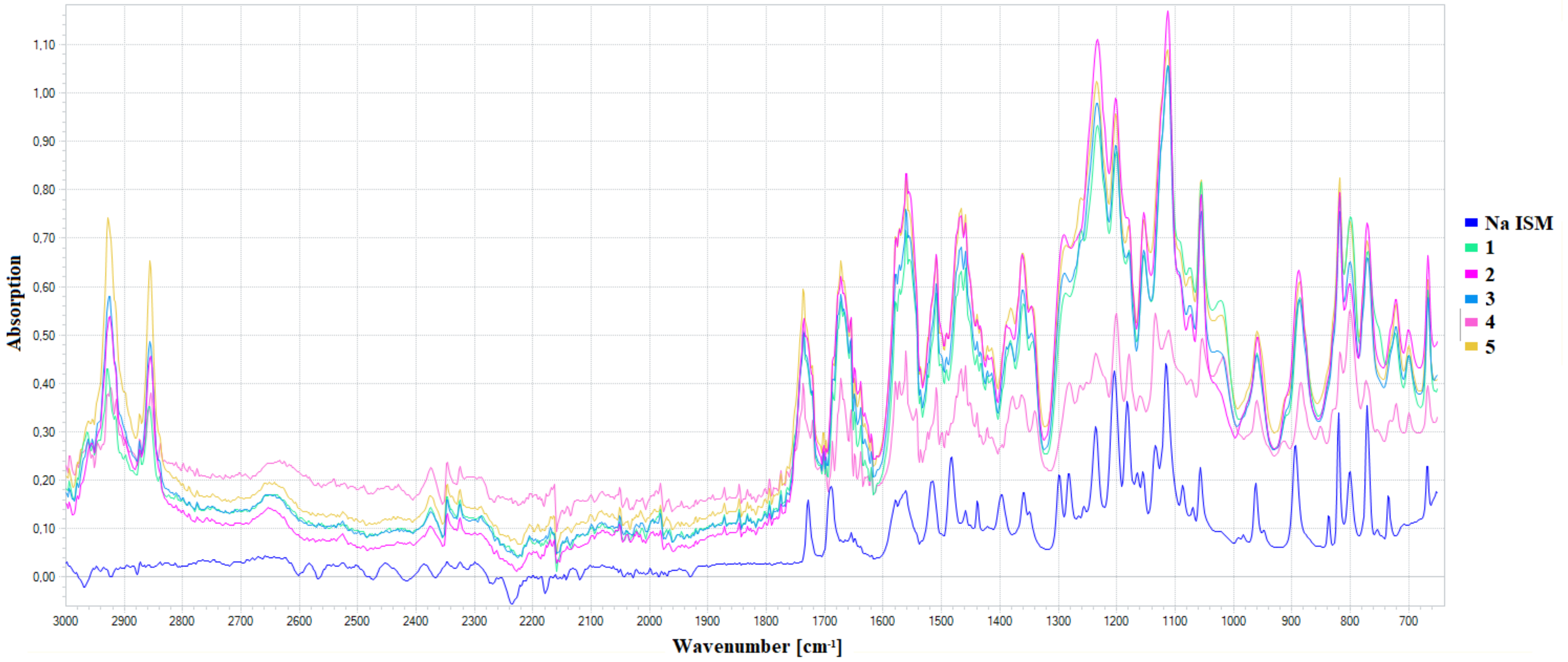

***Figure S35**. The comparison between FT-IR spectra of products with dialkyldimethylammonium cation (**6-8**) compared to iodosulfuron-methyl sodium salt **[Na][ISM]**.*

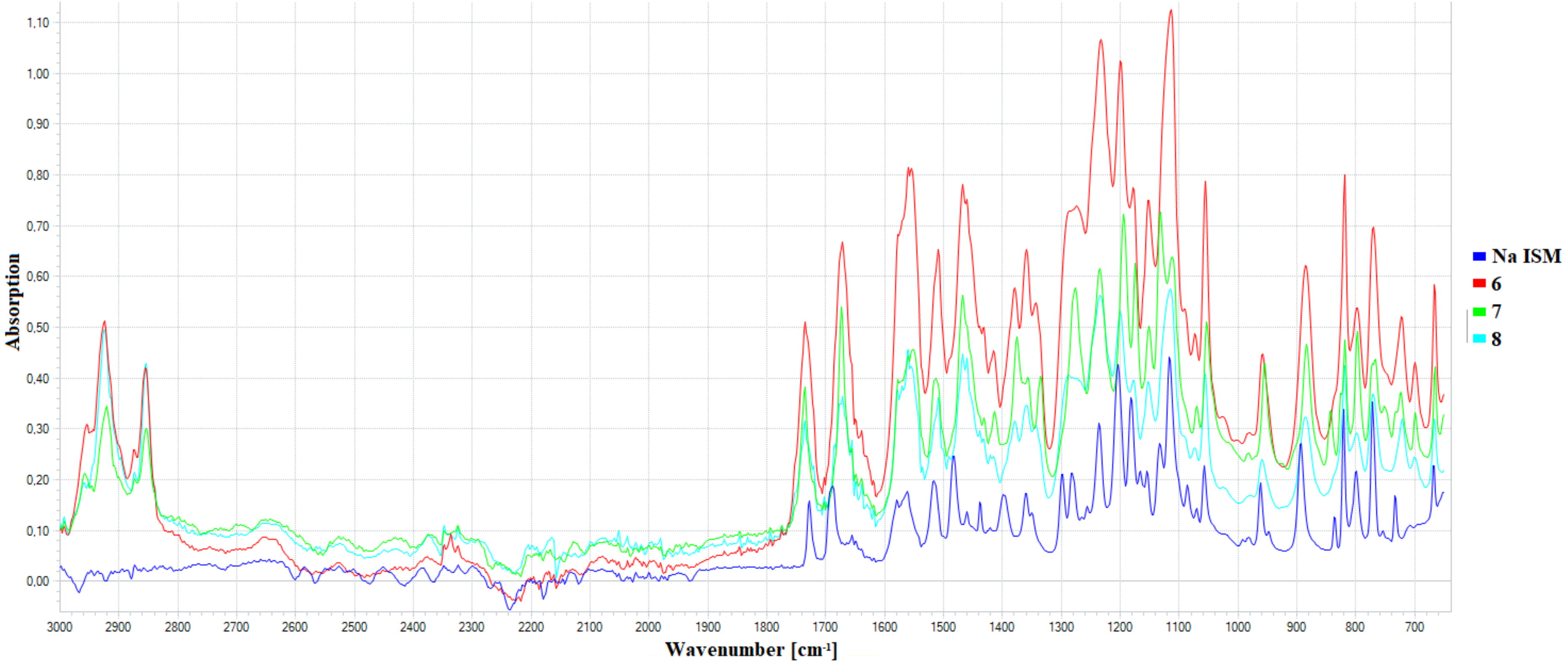

Table S1. The interaction energies of the cation with the anion in the substrates and obtained ILs.

Substrate	E_w [kcal mol^{-1}]	Product - IL	E_w [kcal mol^{-1}]
**S1** (with C_8 alkyl)[a]	109.07	**1** (with C_8 alkyl)[a]	81.37
**S1** (with C_{10} alkyl)[a]	109.07	**1** (with C_{10} alkyl)[a]	81.35
**S1** (with C_{12} alkyl)[a]	108.48	**1** (with C_{12} alkyl)[a]	81.35
**S2**	89.87	**2**	81.97
**S3**	109.07	**3**	81.88
**S4**	108.05	**4**	80.79
**S5**	105.14	**5**	82.30
**S6**	107.55	**6**	81.48
**S7**	107.58	**7**	81.70

[a] calculations were performed for compounds with only one defined alkyl substituent

Table S2. Analysis of FT-IR spectrum of the product with mirystyltrimethylammonium cation **2**

No	Wavenumber [cm^{-1}]	Vibrations	No	Wavenumber [cm^{-1}]	Vibrations
1	2925	C-Hγ alif.	***11***	1345	C-Nγ arom.
2	2855	C-Hγ alif.	***12***	1290	C-Oγ ester
3	1736	C=Oγ ester	***13***	1233	Ph-O-Cγ alif.
4	1679	C=Oγ amide	***14***	1202	C-O-Phγ alif.
5	1562	N-Hσ	***15***	1113	S=Oγ sym.
6	1562	C=Cγ arom	***16***	1055	C-Hγ arom in plane
7	1510	C=Nγ arom	***17***	887	C-Hγ arom.out of plane
8	1467	C-Hσ alif.	***18***	819	C-Hγ arom.out of plane
9	1417	C-Nγ alif.	***19***	773	C=Cσ
10	1388	S=Oγ asym			

Table S3. Analysis of FT-IR spectra of all products compared to sodium salt of iodosulfuron-methyl **[Na][ISM]**

No	Vibrations	Compound - wavenumber [cm^{-1}]								
		**1**	**2**	**3**	**4**	**5**	**6**	**7**	**8**	**[Na][ISM]**
1	C-Hγ alif.	2930	2925	2927	2923	2928	2924	2921	2924	---
2	C-Hγ alif.	2855	2855	2855	2853	2855	2854	2854	2856	---
3	C=Oγ ester	1738	1736	1738	1738	1738	1735	1735	1735	1730
4	C=Oγ amide	1672	1679	1672	1671	1671	1672	1674	1672	1692
5	N-Hσ	1656	1655	1655	1655	1655	1656	1656	1656	1653
6	C=Cγ arom	1563	1562	1563	1562	1562	1560	1561	1561	1562
7	C=Nγ arom	1511	1510	1511	1510	1510	1509	1514	1510	1520
8	C-Hσ alif.	1468	1467	1468	1467	1467	1468	1467	1467	1485
9	C-Nγ alif.	1422	1417	1421	1421	1421	1431	1431	1431	1439
10	S=Oγ asym	1361	1388	1362	1365	1362	1359	1357	1359	1362
11	C-Nγ arom.	1343	1345	1346	1340	1344	1343	1337	1343	1347
12	C-Oγ ester	1284	1290	1291	1281	1261	1274	1278	1289	1282
13	Ph-O-Cγ alif.	1232	1233	1234	1236	1234	1233	1235	1235	1236
14	C-O-Phγ alif.	1200	1202	1203	1200	1202	1200	1195	1200	1207
15	S=Oγ sym.	1110	1113	1112	1111	1113	1113	1110	1116	1117
16	C-Hγ arom in plane	1055	1055	1055	1054	1055	1056	1053	1054	1059
17	C-Hγ arom.out of plane	888	887	887	884	885	886	883	885	895
18	C-Hγ arom.out of plane	819	819	819	819	819	819	818	818	822
19	C=Cσ	771	773	772	771	772	770	772	770	772

Spectral analysis

Due to the presence of two aromatic rings, in a region of 190-400 nm all the products possessed two characteristic maxima (λ_{max}) at approx. 200 (E2 band) and 235 nm (B band) in water (Figs. S1, S5, S9, S13, S17, S21, S25, S29). Similar λ_{max} were noted for triazine derivatives that are substituted with phenyl ring.[1] The calculated values of molar absorptivities ($\varepsilon_{\lambda max}$) that occurred at the highest peak (235 nm) were in a range from 2.24 $\cdot 10^4$ M^{-1} cm^{-1} for **6** to 2.58 $\cdot 10^4$ M^{-1} cm^{-1} for **3**. These results are lower than those of the iodosulfuron-methyl sodium salt ($\varepsilon_{\lambda max}$ = 2.91 $\cdot 10^4$ M^{-1} cm^{-1}), which means that the presence of bulky organic cations led to reduction of this parameter. Additionally, the previous report describing UV spectrum of iodosulfuron-methyl sodium salt in methanol indicate that the solvent has insignificant impact on the location of absorption maximum as well as the molar absorptivity (in methanol: λ_{max} = 239 nm; $\varepsilon_{\lambda max}$ = 2.91 $\cdot 10^4$ M^{-1} cm^{-1}).[2]

In the FT-IR spectra of ILs (Figs. S2, S6, S10, S14, S18, S22, S26, S30) we can distinguish strong peaks originating from the iodosulfuron-methyl anion as well as the cations. The analysis of the most characteristic signals for IL with mirystyltrimethylammonium cation (**2**) is provided in supporting information in Fig. S33 and Table S1. Analogously, the location of these peaks was listed for all products in Table S2 (ESI). The most essential signals can be attributed to stretching vibrations from the amide group ($C{=}O_{\gamma\ amide}$) at approx. 1670 cm^{-1} or the signal from the ester substituent ($C{=}O_{\gamma\ ester}$) at 1740 cm^{-1}. We can also observe other characteristic bands, originating from the conjugated stretching vibrations from aromatic ring ($C{=}C\gamma_{\ arom}$) at approx. 1560 cm^{-1} or stretching vibrations from triazine ring ($C{=}N\gamma_{\ arom}$) at about 1510 cm^{-1}.[3] Signals at 1360-1380 cm^{-1} are due to asymmetric vibrations of the sulfonamide group ($S{=}O\gamma_{\ asym}$), while S=O symmetrical stretching vibrations ($S{=}O\gamma_{\ sym}$) occurred at approx. 1110 cm^{-1}, which corresponds well with the available literature data.[4] Interestingly, the comparison of the FT-IR spectra of the products (comprising alkyltrimethylammonium cations (**1**-**5**) - Fig. S34, comprising dialkyldimethylammonium cations (**6**-**8**) - Fig. S35, ESI) revealed a successive growth of the intensity of the bands at 2800-3000 cm^{-1} in relation to $C{=}O_{\gamma}$ vibrations (1650-1750 cm^{-1}) as the length of the alkyl substituents in cations is increased. This confirms the presence of organic tetraalkylammonium cation in the structures of the products.

The ^1H NMR analysis (Figs. S3, S7, S11, S15, S19, S23, S27, S31) revealed that signals originating from iodosulfuron-methyl anion appeared as singlets at approx. 2.4 ppm (protons from the methyl group) and 3.9 ppm, protons from the methyl in the ether and ester groups). Three protons from the aromatic ring were noted as: doublet at approx. 7.1 ppm, multiplet at 7.7 ppm and singlet at 8.4 ppm, respectively. Interestingly, the mentioned multiplet at 7.7 ppm encompassed also signal from the proton of the amide group, however, it can be even invisible in the spectra of sulfonylureas in the case of utilization of polar protic solvents, like deuterated

methanol (CD_3OD).[2] According to collected data, the signals from the long alkyls in utilized cations appeared at approx. 0.9 ppm (triplet), 1.3 ppm (multiplet), 1.6 ppm (multiplet) 3.2 ppm (multiplet), whereas peaks from the methyl groups attached to nitrogen atom occurred as singlet at approx. 3.2 ppm.

In the ^{13}C NMR spectra of the products (Figs. S4, S8, S12, S16, S20, S24, S28, S32) signals from triazine ring occurred at the greatest values (169-179 ppm), while from benzene ring in a range from 95 to 144 ppm (the lowest value can be attributed to carbon substituted with iodine atom). Other characteristic peaks were found at 165 and 155 ppm, which originate from carbons from ester and amide group, respectively. Due to the presence of long alkyl chains all the products possessed multiple signals in a region beneath 35 ppm. Carbons attached directly to positively charged nitrogen atom were noted at 53 (methyl groups) and 67 ppm (methylene group), accordingly.

Reference for spectral analysis:

1. Koopman, H. U.V. spectra of derivatives of 1,3,5-triazine. *Recl. des Trav. Chim. des Pays-Bas* **1961**, *80*, 158–172, doi:10.1002/recl.19610800206.
2. Niemczak, M.; Sobiech, Ł.; Grzanka, M. Iodosulfuron-Methyl-Based Herbicidal Ionic Liquids Comprising Alkyl Betainate Cation as Novel Active Ingredients with Reduced Environmental Impact and Excellent Efficacy. *J. Agric. Food Chem.* **2020**, *68*, 13661–13671, doi:10.1021/acs.jafc.0c05850.
3. Shurvell, H.F.; Faniran, J.A.; Dodsworth, H. The infrared spectrum of 2,4,6-triphenyl-d15-1,3,5-triazine. *Spectrochim. Acta Part A Mol. Spectrosc.* **1967**, *23*, 1313–1318, doi:10.1016/0584-8539(67)80352-0.
4. Gowda, B.T.; Jyothi, K.; D'Souza, J.D. Infrared and NMR Spectra of Arylsulphonamides, 4-X-C6H4SO2NH2 and i-X, j-YC6H3SO2NH2 (X=H; CH3; C2H5; F; Cl; Br; I or NO2 and i-X, j-Y=2,3-(CH3)2; 2,4-(CH3)2; 2,5- (CH3)2; 2-CH3, 4-Cl; 2-CH3, 5-Cl; 3-CH3, 4-Cl; 2,4-Cl2 or 3,4-Cl2). *Zeitschrift fur Naturforsch. - Sect. A J. Phys. Sci.* **2002**, *57*, 967–973, doi:10.1515/zna-2002-1210.

Table S4. Values of water solubility for ILs **1-8** and sodium salt of iodosulfuron-methyl **[Na][ISM]** at 25 °C.

	**Compound**								
	**1**	**2**	**3**	**4**	**5**	**6**	**7**	**8**	**[Na][ISM]**
**Solubility [g dm^{-3}]**	53.35	2.43	3.57	0.02	0.01	0.19	0.18	0.05	28.75
**SD[a]**	1.420	0.078	0.022	0.003	0.001	0.020	0.012	0.004	1.490

[a] standard deviation

Table S5. Values of logarithm of octanol-water partition coefficient for ILs **1-8** and sodium salt of iodosulfuron-methyl **[Na][ISM]** at 25 ℃.

Salt	Log Kow	SD[a]
**1**	0.201	0.006
**2**	0.513	0.009
**3**	0.514	0.007
**4**	0.518	0.009
**5**	0.534	0.017
**6**	0.452	0.050
**7**	0.607	0.010
**8**	0.672	0.010
**[Na][ISM]**	-1.260	0.022

[a] standard deviation

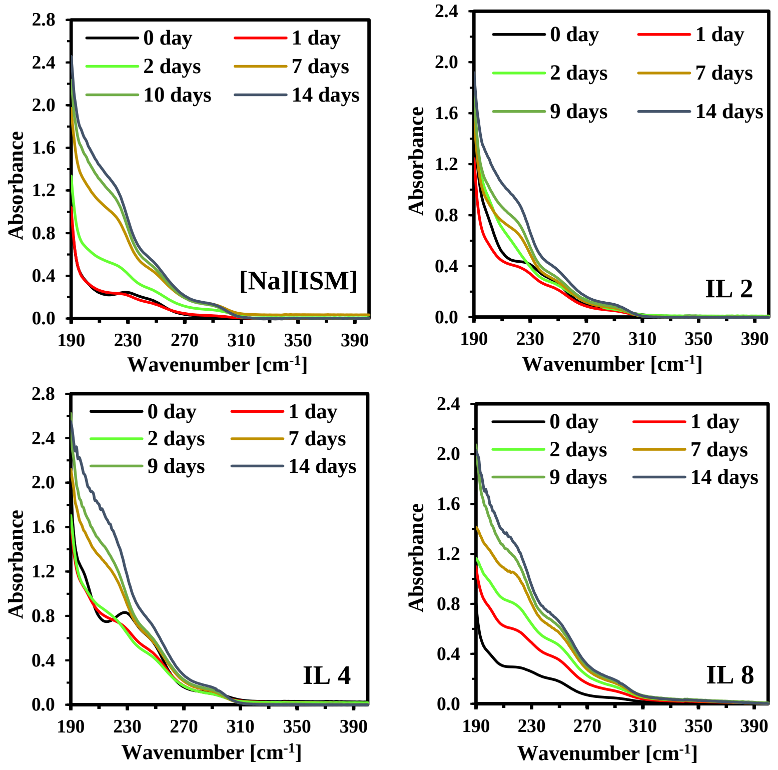

Figure S36. UV spectra of iodosulfuron-methyl sodium salt (**[Na][ISM]**) and ILs **2-4** and **8** in acidic environment.

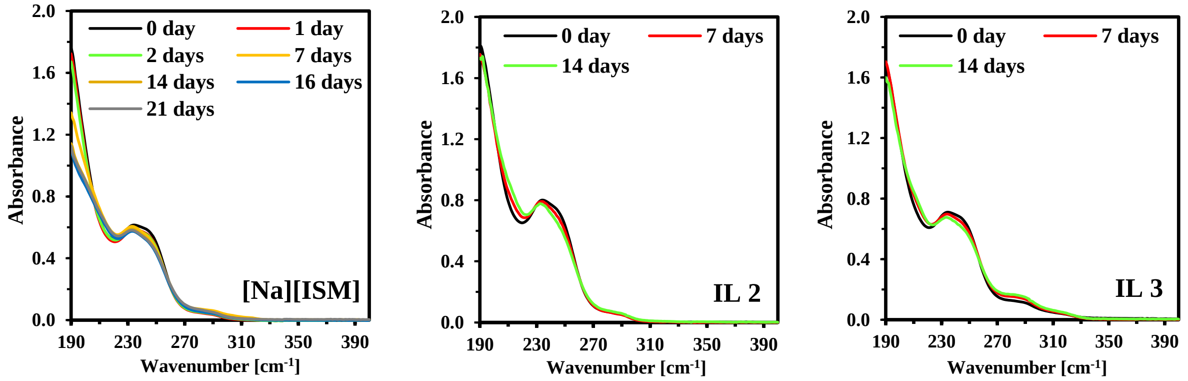

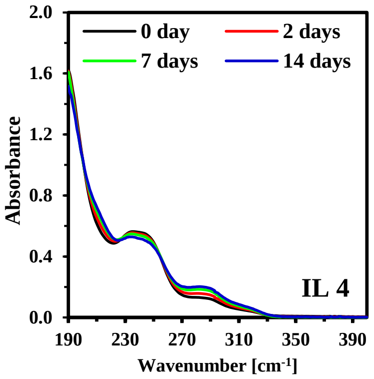

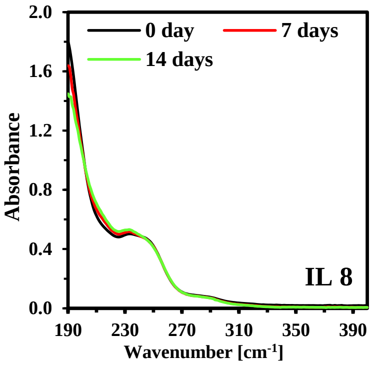

Figure S37. UV spectra of iodosulfuron-methyl sodium salt (**[Na][ISM]**) and ILs **2-4** and **8** in basic environment.

Table S6. Volatility of the studied compounds at 35 ℃.

**Salt**	**Mass of emitted VOC [%]**
**1**	0.02
**2**	0.00
**3**	0.02
**4**	0.00
**5**	0.06
**6**	0.03
**7**	0.92
**8**	0.02
**REF**[a]	0.00

[a] [Na][ISM]

Table S7. Efficacy of the prepared ILs (**1-8**) toward cornflower (*Centaurea cyanus* L.) and oil-seed rape (*Brassica napus* L.)

	Cornflower		Oil-seed rape	
	Fresh weight reduction [%]	SEM [%]	Fresh weight reduction [%]	SEM [%]
**1**	24.27 b	7.28	27.18 b	11.55
**2**	35.00 b	3.34	49.22 a	6.23
**3**	46.24 a	4.36	31.16 b	5.03
**4**	38.40 ab	6.99	35.83 ab	11.24
**5**	12.88 c	0.43	11.71 b	9.57
**6**	16.66 bc	4.77	11.76 c	1.69
**7**	28.49 b	3.81	31.46 b	1.51
**8**	39.10 ab	2.81	36.00 ab	12.55
**Ref**	32.93 b	1.23	40.24 ab	2.71

Ref - HUZAR 05 WG containing sodium salt of iodosulfuron-methyl and adjuvants (heavy aromatic hydrocarbons and unspecified petroleum fraction)
SEM - Standard error of mean
All tested compounds were used in a dose equal to 7.5 g of active ingredient (anion) per hectare
Different letters in columns indicate statistically important differences ($p<0.05$); a - the highest efficacy of the plant protection product

Table S8. Migration of HILs in agricultural soil (soil TLC analysis)

Salt	Rf ± SD
**1**	9.8 ± 0.02
**2**	9.3 ± 0.01
**3**	0.01 - 9.6 ± 0.01[b]
**4**	0.01 - 9.2 ± 0.02[b]
**5**	0.01 - 9.4 ± 0.01[b]
**6**	9.8 ± 0.01
**7**	9.6 ± 0.02
**8**	9.6 ± 0.02
**REF[a]**	9.6 ± 0.01

[a] [Na][ISM]; [b] compound was evenly spread along the whole length of the TLC plate
